# Effect of Colchicine vs Standard Care on Cardiac and Inflammatory Biomarkers and Clinical Outcomes in Patients Hospitalized With Coronavirus Disease 2019: The GRECCO-19 Randomized Clinical Trial

**DOI:** 10.1001/jamanetworkopen.2020.13136

**Published:** 2020-06-24

**Authors:** Spyridon G. Deftereos, Georgios Giannopoulos, Dimitrios A. Vrachatis, Gerasimos D. Siasos, Sotiria G. Giotaki, Panagiotis Gargalianos, Simeon Metallidis, George Sianos, Stefanos Baltagiannis, Periklis Panagopoulos, Konstantinos Dolianitis, Efthalia Randou, Konstantinos Syrigos, Anastasia Kotanidou, Nikolaos G. Koulouris, Haralampos Milionis, Nikolaos Sipsas, Charalampos Gogos, George Tsoukalas, Christoforos D. Olympios, Eleftheria Tsagalou, Ilias Migdalis, Styliani Gerakari, Christos Angelidis, Dimitrios Alexopoulos, Pericles Davlouros, George Hahalis, Ioannis Kanonidis, Demosthenes Katritsis, Theofilos Kolettis, Antonios S. Manolis, Lampros Michalis, Katerina K. Naka, Vlasios N. Pyrgakis, Konstantinos P. Toutouzas, Filippos Triposkiadis, Konstantinos Tsioufis, Emmanouil Vavouranakis, Luis Martinèz-Dolz, Bernhard Reimers, Giulio G. Stefanini, Michael Cleman, John Goudevenos, Sotirios Tsiodras, Dimitrios Tousoulis, Efstathios Iliodromitis, Roxana Mehran, George Dangas, Christodoulos Stefanadis, on behalf of the GRECCO-19 investigators

**Affiliations:** 1 Second Department of Cardiology, Attikon Hospital, National and Kapodistrian University of Athens, Athens, Greece; 2 Department of Cardiology, G. Gennimatas General Hospital of Athens, Athens, Greece; 3 Cardio Center, Humanitas Clinical and Research Hospital IRCCS, Rozzano-Milan, Italy; 4 First Department of Cardiology, Hippokration Hospital, National and Kapodistrian University of Athens, Athens, Greece; 5 Athens Medical Center, Athens, Greece; 6 First Department of Internal Medicine, AHEPA Hospital, Aristotle University of Thessaloniki, Thessaloniki, Greece; 7 First Department of Cardiology, AHEPA Hospital, Aristotle University of Thessaloniki, Thessaloniki, Greece; 8 Department of Internal Medicine, General Hospital of Kastoria, Kastoria, Greece; 9 Second Department of Internal Medicine, General Hospital of Alexandroupoli, Democritus University of Thrace, Alexandroupoli, Greece; 10 Department of Internal Medicine, Mpodosakio General Hospital of Ptolemaida, Ptolemaida, Greece; 11 Department of Internal Medicine, General Hospital of Kozani, Kozani, Greece; 12 Third Department of Internal Medicine, General Hospital Sotiria, National and Kapodistrian University of Athens, Athens, Greece; 13 First Intensive Care Unit, General Hospital Evangelismos, National and Kapodistrian University of Athens, Athens, Greece; 14 First Department of Pneumonology, General Hospital Sotiria, National and Kapodistrian University of Athens, Athens, Greece; 15 First Department of Internal Medicine, Ioannina University Hospital, University of Ioannina, Ioannina, Greece; 16 Infectious Diseases Unit, Laiko General Hospital, Athens, Greece; 17 Internal Medicine Department, University Hospital of Patras, Patras, Greece; 18 Fourth Department of Pneumonology, General Hospital Sotiria, Athens, Greece; 19 Department of Cardiology, General Hospital of Elefsina Thriasio, Elefsina, Greece; 20 Therapeutics Department, Alexandra Hospital, Athens, Greece; 21 Second Medical Department, NIMTS Hospital, Athens, Greece; 22 Department of Internal Medicine, General Hospital of West Attica Agia Varvara, Athens, Greece; 23 Department of Cardiology, University of Patras Medical School, Patras, Greece; 24 Second Department of Cardiology, AHEPA Hospital, Aristotle University of Thessaloniki, Thessaloniki, Greece; 25 Third Department of Cardiology, Hygeia Hospital, Athens, Greece; 26 Department of Cardiology, Ioannina University Hospital, University of Ioannina, Ioannina, Greece; 27 Department of Cardiology, University General Hospital of Larissa, Larissa, Greece; 28 Third Department of Cardiology, General Hospital Sotiria, National and Kapodistrian University of Athens, Athens, Greece; 29 Hospital Universitario y Politécnico La Fe, Valencia, Spain; 30 Section of Cardiovascular Medicine, Yale University School of Medicine, New Haven, Connecticut; 31 Fourth Department of Internal Medicine, Attikon Hospital, National and Kapodistrian University of Athens, Athens, Greece; 32 Icahn School of Medicine at Mount Sinai, New York, New York

## Abstract

**Importance:**

Severe acute respiratory syndrome coronavirus 2 infection has evolved into a global pandemic. Low-dose colchicine combines anti-inflammatory action with a favorable safety profile.

**Objective:**

To evaluate the effect of treatment with colchicine on cardiac and inflammatory biomarkers and clinical outcomes in patients hospitalized with coronavirus disease 2019 (COVID-19).

**Design, Setting, and Participants:**

In this prospective, open-label, randomized clinical trial (the Greek Study in the Effects of Colchicine in COVID-19 Complications Prevention), 105 patients hospitalized with COVID-19 were randomized in a 1:1 allocation from April 3 to April 27, 2020, to either standard medical treatment or colchicine with standard medical treatment. The study took place in 16 tertiary hospitals in Greece.

**Intervention:**

Colchicine administration (1.5-mg loading dose followed by 0.5 mg after 60 min and maintenance doses of 0.5 mg twice daily) with standard medical treatment for as long as 3 weeks.

**Main Outcomes and Measures:**

Primary end points were (1) maximum high-sensitivity cardiac troponin level; (2) time for C-reactive protein to reach more than 3 times the upper reference limit; and (3) time to deterioration by 2 points on a 7-grade clinical status scale, ranging from able to resume normal activities to death. Secondary end points were (1) the percentage of participants requiring mechanical ventilation, (2) all-cause mortality, and (3) number, type, severity, and seriousness of adverse events. The primary efficacy analysis was performed on an intention-to-treat basis.

**Results:**

A total of 105 patients were evaluated (61 [58.1%] men; median [interquartile range] age, 64 [54-76] years) with 50 (47.6%) randomized to the control group and 55 (52.4%) to the colchicine group. Median (interquartile range) peak high-sensitivity cardiac troponin values were 0.0112 (0.0043-0.0093) ng/mL in the control group and 0.008 (0.004-0.0135) ng/mL in the colchicine group (*P* = .34). Median (interquartile range) maximum C-reactive protein levels were 4.5 (1.4-8.9) mg/dL vs 3.1 (0.8-9.8) mg/dL (*P* = .73), respectively. The clinical primary end point rate was 14.0% in the control group (7 of 50 patients) and 1.8% in the colchicine group (1 of 55 patients) (odds ratio, 0.11; 95% CI, 0.01-0.96; *P* = .02). Mean (SD) event-free survival time was 18.6 (0.83) days the in the control group vs 20.7 (0.31) in the colchicine group (log rank *P* = .03). Adverse events were similar in the 2 groups, except for diarrhea, which was more frequent with colchicine group than the control group (25 patients [45.5%] vs 9 patients [18.0%]; *P* = .003).

**Conclusions and Relevance:**

In this randomized clinical trial, participants who received colchicine had statistically significantly improved time to clinical deterioration. There were no significant differences in high-sensitivity cardiac troponin or C-reactive protein levels. These findings should be interpreted with caution.

**Trial Registration:**

ClinicalTrials.gov Identifier: NCT04326790

## Key Points

**Question:**

Is the receipt of colchicine among patients hospitalized with symptomatic coronavirus disease 2019 associated with clinical benefit?

**Findings:**

In this randomized clinical trial of 105 patients, the rate of the primary clinical end point (clinical deterioration) was higher in the control group than in the colchicine group, and the time to clinical deterioration was shorter in the control group than in the colchicine arm. No difference was observed in the primary biochemical end point (high-sensitivity troponin concentration), but patients in the colchicine group had a smaller increase in dimerized plasma fragment D compared with patients in the control group.

**Meaning:**

The hypothesis-generating findings of this study suggest a role for colchicine in the treatment of patients with coronavirus disease 2019.

## Teaser

This randomized clinical trial evaluates the effect of treatment with colchicine on cardiac and inflammatory biomarkers and clinical outcomes in patients hospitalized with coronavirus disease 2019 (COVID-19).

## Introduction

Severe acute respiratory syndrome coronavirus 2 (SARS-CoV-2) infection evolved into a global pandemic during the first quarter of 2020. Early data revealed that coronavirus disease 2019 (COVID-19) results in an intense inflammatory response predominantly affecting the respiratory system, leading to acute lung injury or acute respiratory distress syndrome in certain cases.^1^

Within this pathophysiologic framework, potentially successful treatment candidates should possess potent anti-inflammatory action without the adverse effects of steroids and nonsteroidal anti-inflammatory agents. Colchicine has been traditionally used to treat gout and rheumatic disease, and in recent years, a number of studies have shown a favorable safety-benefit profile among patients with cardiovascular disease, including pericarditis.^2^ The anti-inflammatory action of colchicine is mediated by completely different pathophysiologic routes than that of corticosteroids and nonsteroidal anti-inflammatory agents. In addition, colchicine inhibits neutrophil chemotaxis and activity in response to vascular injury, inhibits inflammasome signaling and reduces the production of active interleukin-1β, reduces neutrophil-platelet interaction and aggregation, and has rapid onset of anti-inflammatory effects when the standard oral regimen of colchicine used for gout flares (1.2 mg initially followed by 0.6 mg every hour for 6 hours or until severe gastrointestinal symptoms occur) is followed.^3^

These features suggest that colchicine might combine anti-inflammatory action with an acceptable safety profile, leading to the hypothesis that it might be a safe and effective anti-inflammatory treatment choice for COVID-19.^4,5,6^ In the present prospective, randomized, multicenter clinical trial, we sought to evaluate the potential of colchicine to improve outcomes among patients hospitalized with COVID-19.

## Methods

### Trial Design and Oversight

**Figure 1.**
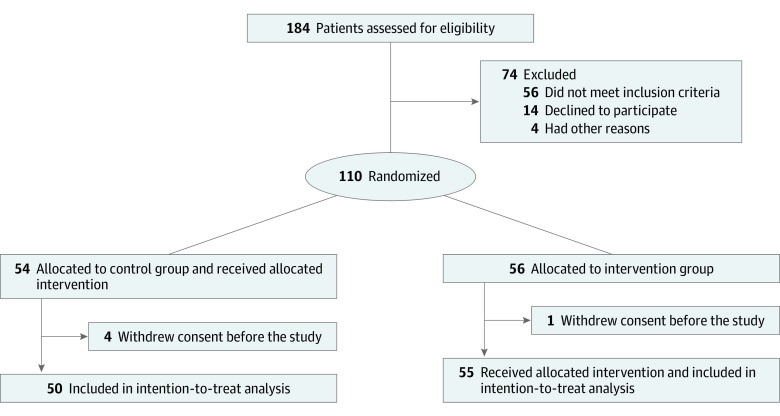
Study Flow Diagram

In this prospective, open-label, randomized clinical trial, we assigned patients in a 1:1 allocation to receive either optimal medical treatment according to local protocols, as established by the National Public Health Organization^7^ and following the guidance of the European Centre for Disease Prevention and Control (control group) or colchicine in addition to optimal medical treatment (intervention group) (Figure 1). The detailed trial protocol is available in Supplement 1, and the study rationale has been published previously.^6^ The protocol was approved by the National Ethics Committee and the Hellenic National Organization for Medicines. All patients provided written informed consent before enrollment. The trial was reported according to Consolidated Standards of Reporting Trials (CONSORT) reporting guideline.^8^

A total of 16 tertiary care hospitals in Greece were activated for patient recruitment. Eligible patients were randomly assigned (1:1) to either the control group or the colchicine group. The randomization sequence was prepared by a statistician not involved in the trial using R software version 3.6.2 (R Project for Statistical Computing), and the corresponding assignment was provided to site coordinators electronically on each patient enrollment.

### Trial Population

Hospitalized adult patients diagnosed with SARS-CoV-2 infection, confirmed with polymerase chain reaction–reverse transcriptase testing, were considered eligible if they had a body temperature of 37.5 °C or greater and 2 or more of the following: sustained coughing, sustained sore throat, anosmia and/or ageusia, fatigue and/or tiredness, and arterial oxygen partial pressure lower than 95 mm Hg on room air. Exclusion criteria included pregnancy or lactation, known hypersensitivity to colchicine, known hepatic failure, estimated glomerular filtration rate under 20 mL/min/1.73 m^2^, corrected QT interval of 450 milliseconds or higher (according to the Bazett formula) on a 12-lead surface electrocardiogram (although colchicine has no known effects on repolarization, this exclusion criterion was implemented because of concerns that a potential interaction between colchicine and hydroxychloroquine could lead to excess QT prolongation caused by the latter), or clinical assessment indicating that ventilatory support would be inevitable in the following 24 hours because of rapidly declining respiratory status. A comprehensive description of eligibility and exclusion criteria is provided in the study protocol in Supplement 1.

### Trial Procedures

In addition to other medical treatment for COVID-19 per local protocols, patients who were randomized to the intervention arm received a loading dose of colchicine followed by maintenance daily dosage. The loading dose consisted of 1.5 mg of colchicine followed by 0.5 mg of colchicine 60 minutes later if no adverse gastrointestinal effects were observed. In the case of azithromycin coadministration, a single 1.0-mg loading dose of colchicine was administered. The maintenance dosage was 0.5 mg colchicine twice daily (reduced to once daily among patients with body weight <60 kg) until hospital discharge or a maximum of 21 days. All patients were observed during this time frame with daily reassessment of their clinical status and complete laboratory hematologic and biochemical evaluation every 48 hours.

### End Points

Study end point analysis was planned to be performed in 2 phases, an early biochemical phase and a later clinical phase. The coprimary end points of the biochemical phase were the difference in maximal high-sensitivity cardiac troponin (hs cTn) levels between the 2 groups and the time for C-reactive protein to reach levels greater than 3 times the upper reference limit. The primary end point of the clinical phase was the time from baseline to clinical deterioration, defined as a 2-grade increase on an ordinal clinical scale, based on the World Health Organization R&D Blueprint Ordinal Clinical Scale,^9^ as used in previously published studies,^10^ within a time frame of 3 weeks after randomization or until hospital discharge (whichever occurred first). The 7-grade ordinal scale consisted of the following levels: 1, ambulatory, normal activities; 2, ambulatory but unable to resume normal activities; 3, hospitalized, not requiring supplemental oxygen; 4, hospitalized, requiring supplemental oxygen; 5, hospitalized, requiring nasal high-flow oxygen therapy, noninvasive mechanical ventilation, or both; 6, hospitalized, requiring extracorporeal membrane oxygenation, invasive mechanical ventilation, or both; and 7, death. Secondary end points were the percentage of participants requiring mechanical ventilation, all-cause mortality at the end of follow-up, and the number, type, severity, and seriousness of total adverse events and treatment-related adverse events.

### Statistical Analysis

The primary efficacy analysis was performed on an intention-to-treat basis. Assuming a median event-free survival of 30 days and an accrual time of 30 days, it was calculated that 180 patients were needed to have a 90% probability to detect a 50% reduction in the primary clinical end point at α = .05. For the biochemical analysis, it was estimated 85 patients were needed to have a 90% probability to detect a 30% reduction in peak hs cTn level, assuming a median hs cTn level of 0.05 ng/mL (to convert to micrograms per liter, multiply by 1.0) in the control group, at α = .05. Continuous parameters were summarized as median and interquartile range (IQR) and compared with nonparametric tests (Mann-Whitney). The Hodges-Lehmann estimate was used to calculate 95% CIs for the difference between medians. Categorical variables are reported as counts and percentages and were compared with the χ^2^ test. In cases in which the 2 × 2 matrices contained cells with expected values less than 5, the Fisher exact test was used. The Cochran-Armitage test was used to test for trends in 2 × k contingency tables. Odds ratios for the clinical end point were calculated with the Mantel-Haenszel test. Kaplan-Meier analysis was used to assess the time to clinical deterioration, and the log rank test was used to compare end point–free survival between the 2 groups (primary clinical efficacy analysis). No specific statistical handling of missing values was performed. Statistical significance was set at *P* < .05, and all tests were 2-tailed. SPSS statistical software version 25 (IBM Corp) was used for all statistical analyses.

## Results

Patient recruitment started on April 3, 2020, and was terminated on April 27, 2020, because of slow enrollment as a result of the rapid flattening of the curve of COVID-19 cases in Greece. Overall, 105 patients (61 [58.1%] men; median [IQR] age, 64 [54-76] years) fulfilled the admission criteria and were included and randomized at 16 clinical sites (Figure 1). Their baseline characteristics are summarized in Table 1; most patients received chloroquine or hydroxychloroquine (103 [98.1%]) and azithromycin (97 [92.4%]). The 2 treatment groups were largely similar in terms of demographic characteristics, clinical status at presentation, and baseline laboratory evaluation (Table 1). The median (IQR) baseline clinical score was 4 (3-4) in both groups. Median (IQR) hospitalization duration was 12 (9-22) days in the colchicine groups and 13 (9-18) days in the control group (*P* = .91). No patients were lost to follow-up.

**Table 1.** Baseline Characteristics

Characteristic	No. (%)		Difference (95% CI)^a^
	Control group (n = 50)	Colchicine group (n = 55)	
Men	30 (60.0)	31 (56.4)	4 (–15 to 23)
Age, y, median (IQR)	65 (54 to 80)	63 (55 to 70)	1.5 (–5 to 8)
BMI, median (IQR)	27.7 (24.6 to 30.5)	27.3 (25.2 to 30.5)	0.15 (–1.5 to 1.8)
Time from admission to enrollment, median (IQR), d	5 (1 to 8)	3 (0 to 6)	0.5 (–1 to 2)
Clinical status score			
3	17 (34.0)	19 (34.5)	0 (–18 to 18)
4	30 (60.0)	36 (65.5)	–6 (–25 to 13)
5	3 (6.0)	0	6 (–1 to 13)
Lower respiratory tract infection	47 (94.0)	54 (98.2)	–4 (–12 to 4)
Persistent cough	35 (70.0)	34 (61.8)	8 (–10 to 26)
Anosmia or ageusia	7 (14.0)	7 (12.7)	1 (–12 to 14)
Persistent pharyngeal pain	12 (24.0)	11 (20.0)	4 (–12 to 20)
Fatigue	46 (92.0)	46 (83.6)	8 (–4 to 20)
Pao_2_ <95 mm Hg	33 (66.0)	41 (74.5)	–9 (–26 to 8)
Not smoking	26 (52.0)	31 (56.3)	–4 (–23 to 15)
Formerly smoked	15 (30.0)	15 (27.2)	3 (–14 to 20)
Currently smoking	2 (4.0)	4 (7.3)	–3 (–12 to 6)
Arterial hypertension	25 (50.0)	22 (40.0)	10 (–9 to 29)
Diabetes	12 (24.0)	9 (16.4)	8 (–7 to 23)
Dyslipidemia	16 (32.0)	17 (30.9)	1 (–17 to 19)
Coronary artery disease	5 (10.0)	9 (16.4)	–6 (–19 to 7)
Valvulopathy	1 (2.0)	4 (7.3)	–5 (–13 to 3)
Atrial fibrillation	5 (10.0)	6 (10.9)	–1 (–13 to 11)
Chronic obstructive pulmonary disease	2 (4.0)	3 (5.5)	–2 (–10 to 6)
Known immunosuppression	1 (2.0)	3 (5.5)	–3.5 (–11 to 4)
Concomitant COVID-19 treatment^b^			
Chloroquine or hydroxychloroquine	48 (96.0)	55 (100)	–4 (–9 to 1)
Azithromycin	46 (92.0)	51 (92.7)	–1 (–11 to 9)
Lopinavir or ritonavir	19 (38.0)	14 (25.5)	–13 (–5 tο 31)
Tocilizumab	2 (4.0)	2 (3.6)	0 (–8 to 8)
Concomitant anticoagulation medication	26 (52.0)	31 (56.3)	–4 (–23 to 15)
Temperature, median (IQR), °C	37.7 (37.5 to 38.1)	37.7 (37.5 to 38.1)	0 (–0.1 to 0.1)
Pao_2_, median (IQR), mm Hg	80 (73 to 93)	75 (65 to 97)	4.5 (–2 to 11)
Hemoglobin, g/dL	12.8 (11.2 to 14.5)	12.7 (11.5 to 14.1)	0.3 (–0.4 to 1.0)
White blood cell count, median (IQR), /μL	5784 (4173 to 8163)	5480 (4820 to 6830)	–17.5 (–920 to 885)
Neutrophils, %	70.2 (58.6 to 79.9)	67.8 (62.7 to 76.3)	NA
Lymphocytes, %	19.3 (12.6 to 29.2)	23.2 (14.9 to 27.4)	NA
Macrophages, %	8.0 (4.3 to 9.2)	7.6 (6.2 to 8.7)	NA
Eosinophils, %	0.40 (0.15 to 1.28)	0.65 (0.18 to 1.80)	NA
Lymphocyte count, median (IQR), /μL	1079 (806-1500)	1299 (899-1608)	–116.5 (–316 to 83)
Platelet count, median (IQR), ×10^3^/μL	207 (168 to 326)	221 (169 to 327)	–6 (–46 to 34)
Glucose, median (IQR), mg/dL	106 (91 to 126)	100 (88 to 125)	2 (–8 to 12)
Sodium, median (IQR), mEq/L	139 (137 to 141)	138 (136 to 141)	0.5 (–1 to 2)
Potassium, median (IQR), mEq/L	4.1 (3.8 to 4.5)	4.0 (3.8 to 4.5)	0 (–0.2 to 0.2)
Estimated GFR, median (IQR), mL/min/1.73m^2^	93 (64 to 118)	99 (76 to 128)	–11.5 (–28 to 5)
Aspartate aminotransferase, median (IQR), U/L	34 (23 to 52)	30 (21 to 42)	4 (–2 to 10)
Alanine aminotransferase, median (IQR), U/L	35 (18 to 49)	25 (17 to 43)	4.5 (–3 to 12)
Lactate dehydrogenase, median (IQR), U/L	280 (224 to 405)	251 (196 to 350)	30 (–13 to 73)
Creatine phosphokinase, median (IQR), U/L	80 (55 to 133)	80 (49 to 164)	0 (–23 to 23)
C-reactive protein, median (IQR), mg/dL	4.0 (1.2 to 9.5)	3.6 (1.0 to 6.7)	9.5 (–6 to 25)
High-sensitivity cardiac troponin, median (IQR), ng/mL	0.007 (0.0035 to 0.0185)	0.008 (0.004 to 0.0123)	0.0005 (–2.0 to 3.0)
D-dimer, median (IQR), μg/ml	0.60 (0.40 to 1.01)	0.52 (0.28 to 0.94)	0.0815 (–0.0082 to 0.0245)

Abbreviations: BMI, body mass index (calculated as weight in kilograms divided by height in meters squared); COVID-19, coronavirus disease 2019; D-dimer, dimerized plasma fragment D; GFR, glomerular filtration rate; IQR, interquartile range; NA, not applicable; Pao_2_, partial pressure of oxygen.

SI conversion factors: To convert alanine aminotransferase, aspartate aminotransferase, creatine phosphokinase, and lactate dehydrogenase to microkatals per liter, multiply by 0.0167; C-reactive protein to milligrams per liter, multiply by 10.0; D-dimer to nanomoles per liter, multiply 5.476; high-sensitivity cardiac troponin to micrograms per liter, multiply by 1.0; glucose to millimoles per liter, multiply by 0.0555; hemoglobin to grams per liter, multiply by 10.0; lymphocyte and white blood cell count to ×10^9^ per liter, multiply by 0.001; platelet count to ×10^9^ per liter, multiply by 1.0; and potassium and sodium to millimoles per liter, multiply by 1.0.

^a^ Values in 95% CI of the difference represent rate differences for categorical variables and differences of the medians (Hodges-Lehmann estimate) for continuous variables.

^b^ Darunavir or cobicistat, remdesevir, and human interferon a1b were not used for any patient.

**Figure 2.**
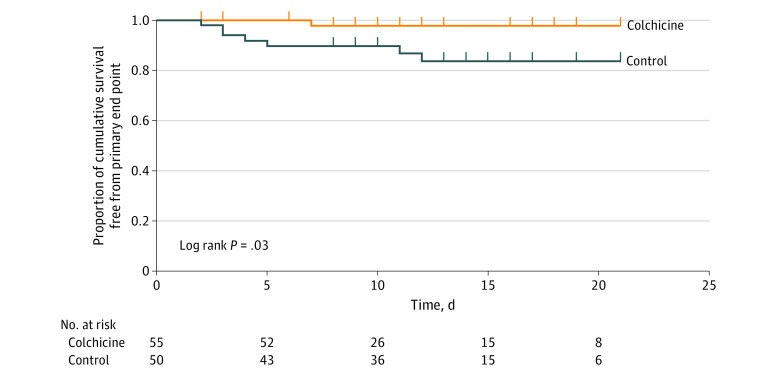
Kaplan-Meier Curves for Survival From the Primary Clinical End Point

The clinical primary end point occurred in 7 patients (14.0%) in the control group and in 1 patient (1.8%) in the colchicine group (*P* = .02), corresponding to a Mantel-Haenszel common odds ratio of 0.11 (95% CI, 0.01-0.96; *P* = .046). Kaplan-Meier event-free survival curves are depicted in Figure 2. Cumulative event-free 10-day survival was 83% vs 97% in the control and colchicine groups, respectively (Gehan statistic, 4.9; *P* = .03). Of the 7 patients who met the primary clinical end point in the control group, 1 (14.3%) needed noninvasive mechanical ventilation (bilevel positive airway pressure), 5 (71.4%) were intubated and ventilated mechanically (3 [42.9%] died shortly after intubation), and 1 (14.3%) died suddenly in the ward of cardiorespiratory arrest. The patient in the colchicine group who met the end point needed invasive mechanical ventilation and died subsequently in the intensive care unit.

The primary outcome measure of the biochemical phase, hs cTn concentration, increased from a median (IQR) of 0.073 (0.037 to 0.0149) ng/mL at baseline to a peak median (IQR) value of 0.0084 (0.0041 to 0.0016) ng/mL, corresponding to a absolute median (IQR) change of 0.0006 (−0.0009 to 0.0042) ng/mL. No significant differences in hs cTn level were observed between the 2 groups at baseline or at peak values (Table 2; eFigure in Supplement 2), with a median (IQR) change from baseline of 0.0011 (−0.0003 to 0.0059) ng/mL in the control group vs 0.0 (−0.001 to 0.0034) ng/mL in the colchicine group (*P* = .26). Peak C-reactive protein levels were also statistically similar in the two groups (Table 1 and Table 2; eFigure in Supplement 2). We do not report results on the prespecified end point of time to reach 3 times the upper reference limit of normal because 72 patients (68.6%) already had CRP values higher than that at baseline (35 [70.0%] in the control group and 37 [67.3%] in the colchicine group; *P* = .76), which does not allow for any meaningful comparison between the 2 groups in terms of this variable.

**Table 2.** Maximum and Minimal Levels of Selected Laboratory Parameters With Potential Prognostic Significance During Treatment

Laboratory parameter	Median (IQR)		Difference (95% CI)^a^	*P* value
	Control group (n = 50)	Colchicine group (n = 55)		
Maximum creatinine phosphokinase, U/L	107 (55 to 179)	105 (55 to 167)	1 (–28 to 30)	.76
Maximum high-sensitivity cardiac troponin, ng/mL	0.0112 (0.0043 to 0.0193)	0.008 (0.004 to 0.0135)	0.0017 (–0.0015 to 0.0049)	.38
Maximum C-reactive protein, mg/dL	4.5 (1.4 to 8.9)	3.1 (0.8 to 9.8)	4.75 (–9.6 to 19.1)	.73
Maximum D-dimer, μg/mL	0.92 (0.68 to 2.77)	0.76 (0.41 to 1.59)	0.3115 (0.01 to 0.613)	.04
Minimum platelet count, ×10^3^/μL	213 (148 to 261)	226 (164 to 296)	-21 (–55 to 13)	.32
Minimum estimated GFR, mL/min/1.73m^2^	86.7 (55.8-114.3)	100.9 (64.2-120.5)	-10 (–25 to 5)	.21
Maximum white blood cell count, /μL	7189 (5490 to 9040)	6366 (5370 to 8240)	672 (–336 to 1680)	.15
Minimum lymphocyte count, /μL	920 (676 to 1293)	1141 (796 to 1477)	156 (–355 to 43)	.11

Abbreviations: D-dimer, dimerized plasma fragment D; GFR, glomerular filtration rate; IQR, interquartile range;

SI conversion factors: To convert creatine phosphokinase to microkatals per liter, multiply by 0.0167; C-reactive protein to milligrams per liter, multiply by 10.0; D-dimer to nanomoles per liter, multiply 5.476; high-sensitivity cardiac troponin to micrograms per liter, multiply by 1.0; lymphocyte and white blood cell count to ×10^9^ per liter, multiply by 0.001; and platelet count to ×10^9^ per liter, multiply by 1.0.

^a^ Values in 95% CI of the difference represent differences of the median (Hodges-Lehmann estimate).

Adverse events were overall similar in the 2 groups (Table 3), with the exception of diarrhea, which was significantly more frequent in the colchicine group than the control group (25 patients [45.5%] vs 9 patients [18.0%]; *P* = .003). However, it was generally self-limited and led to drug discontinuation only in 2 cases (3.6%) (on days 3 and 6). This is also supported by the fact that electrolyte levels did not differ significantly between groups: median (IQR) minimal sodium levels were 137 (133-139) mEq/L in the control group vs 137 (135-139) mEq/L in the colchicine arm (to convert to millimoles per liter, multiply by 1.0) (*P* = .30), and minimal median (IQR) potassium levels were similar, at 3.8 (3.5-4.2) mEq/L vs 3.8 (3.5-4.2) mEq/L (to convert to millimoles per liter, multiply by 1.0), respectively (*P* = .85). Adverse events reported by the field investigators as serious were 1 patient per group (2.0% in the control group and 1.8% in the colchicine group; *P* > .99). However, none of these events (1 case of thrombocytopenia and 1 case of diarrhea, respectively) were serious according to the protocol definition, and both were rated as moderate in severity (per the Common Terminology Criteria for Adverse Events of the National Cancer Institute, ie, noninvasive intervention indicated).

**Table 3.** Adverse Events^a^

Adverse Event	No. (%)		*P* value
	Control group (n = 50)	Colchicine group (n = 55)	
Vomiting	1 (2)	1 (1.8)	>.99
Diarrhea	9 (18)	25 (45.5)	.003
Diarrhea requiring study drug stop	0 (0)	2 (3.6)	.50
Nausea	1 (2)	2 (3.6)	>.99
Abdominal pain	0 (0)	5 (9.1)	.06
Muscle spasm	0 (0)	1 (1.8)	>.99
Headache	1 (2)	1 (1.8)	>.99
Other	3 (6)^b^	6 (10.9)^c^	.50

^a^ All adverse events were graded as mild or moderate in severity.

^b^ Other adverse events included acute renal failure (1 patient [2.0%]), pancytopenia (1 patient [2.0%]), and thrombophlebitis (1 patient [2.0%]).

^c^ These included 1 adverse event (1.8%) related to colchicine (elevated liver enzymes, reversed after colchicine interruption) and 5 of uncertain relation to colchicine (elevated liver enzymes, 1 patient [1.8%]; rhinorrhagia, 1 patient [1.8%]; allergic reaction, 1 patient [1.8%]; cutaneous rash, 1 patient [1.8%]; and chest discomfort, 1 patient [1.8%]).

Peak median (IQR) dimerized plasma fragment D (D-dimer) concentration was significantly lower in the colchicine group than in the control group (0.76 [0.41 to 1.59] μg/mL vs 0.92 [0.68 to 2.77] μg/mL [to convert to nanomoles per liter, multiply by 5.476]; *P* = .04), while baseline levels did not differ significantly (Table 1 and Table 2; eFigure in Supplement 2). D-dimer levels greater than the threshold of 1.00 μg/mL were reported in 24 patients (48.0%) in the control group and 21 patients (38.2%) in the colchicine group (*P* = .32).

Hematological parameters, including leukocyte and platelet counts, were overall similar in the 2 groups both at baseline and during hospitalization, although fewer patients in the colchicine group than the control group had lymphocytopenia (absolute lymphocyte count lower than 1200/μl [to convert to ×10^9^/L, multiply by 0.001]; 32 [58.2%] vs 37 [74.0%]; *P* = .08). A detailed summary of the baseline and peak values of all tested variables is provided in the eTable in Supplement 2.

## Discussion

The findings of the present study suggest a significant clinical benefit from colchicine in patients hospitalized with COVID-19. The definition of clinical deterioration used in this study (2 grades on the ordinal clinical scale) is highly relevant because it essentially translates to a change in clinical condition requiring invasive or noninvasive mechanical respiratory support or death. However, hs cTn levels were not significantly different between groups, while hs cTn increase was, overall, low to modest, far below what cardiologists are used to encountering in patients with acute coronary syndromes or myocarditis.

C-reactive protein levels were not significantly different between the 2 groups. This might be explained by enrollment after a median (IQR) of 3.5 (1-7) days of hospitalization and concurrent administration of other drugs with anti-inflammatory action, such as chloroquine or azithromycin, in both groups.

There was an attenuated D-dimer increase in patients treated with colchicine vs those in the control arm, which suggests an anti-inflammatory and antithrombogenic effect. Although this finding may be a random observation (the reported results are of an exploratory nature, given that D-dimer levels were not part of the initial list of study end points), studies among patients with familial Mediterranean fever^11^ suggest that colchicine may in fact have such an effect; it may be mediated by colchicine anti-inflammatory properties on the endothelium.^3,12^ This may be especially relevant in patients with COVID-19, considering that elevated D-dimer levels have been reported in patients with an unfavorable prognosis,^13,14^ and the activation of hypoxia-responsive signaling pathways leading to increased thrombogenicity have been suggested as plausible mechanisms for these effects.^15^ Subsequent to the beginning of our study, international observations of COVID-19–related multiorgan prothrombotic effects emerged,^14^ and antithrombotic (or even thrombolytic therapy) was advocated in many countries. In our study population, no thrombolytic therapy was used, and antithrombotic therapy was similar in the 2 study groups.

The primary biomarker aim of the present study was to explore the potential of colchicine to attenuate COVID-19–related myocardial injury. An explanation of myocardial injury in COVID-19 is a relative oxygen supply and demand mismatch, direct effects of viral myocarditis and vasculitis, stress-cardiomyopathy, and/or increased sympathetic activity, microthrombotic arterial or venous abnormalities (related to the aforementioned hypercoagulable state), or even de novo acute coronary syndromes due to coronary plaque destabilization in the context of an inflammatory environment.^5^ Low-dose colchicine has been drawing research interest as a potential anti-inflammatory treatment, combining cardiovascular safety with a favorable overall risk-benefit profile, which could reduce myocardial injury in a number of clinical settings.^16,17^ However, in our cohort, maximum levels of hs cTn were comparable in the 2 groups, and the observed magnitude of hs cTn increases was relatively low (ie, mostly within the reference range). This probably indicates that although hs cTn may be considered a marker of adverse outcomes,^18^ myocardial injury itself may not be a major determinant of the clinical course in most patients with COVID-19. Nevertheless, it cannot be precluded that colchicine could show a cardioprotective effect in specific subsets of patients with COVID-19 (eg, those with overt myocardial involvement), but this remains to be explored in other studies. Like the present study, the 2020 COLCHICINE-PCI^19^ trial reported no major effect of colchicine on measures of myocardial injury or inflammatory biomarkers after percutaneous coronary intervention. Finally, international reports indicate very low levels of cardiac events in the era of COVID-19 pandemic.^20^

Considering this information, the observed clinical benefit from colchicine in the present study may be explained by a number of underlying mechanisms. SARS-CoV-2 has been recognized to be highly homologous to SARS-CoV in genome, while both coronaviruses use angiotensin-converting enzyme 2 receptors as entry receptors for the viral spike (S) protein.^21,22^ Furthermore, there is evidence suggesting that SARS-CoV proteins, such as viroporins E, 3a, and 8A, play a crucial role in viral replication and the pathogenetic sequelae thereof.^23^ Therefore, factors that could potentially affect clathrin-mediated endocytosis (a procedure that is partially mediated by microtubules remodeling^24^) could potentially decelerate the viral infection of cells.^25^ In addition, SARS-CoV infection has been implicated in NLRP3 inflammasome activation.^25,26,27,28^ The latter has been shown to be involved in the pathophysiologic cascade of acute lung injury and/or acute respiratory distress syndrome.^29,30,31,32^ Colchicine has been thought to exert its actions mainly through the inhibition of microtubule polymerization and leucocyte infiltration, but it is now presumed that a significant part of colchicine’s anti-inflammatory action is attributed to inhibition of the NLRP3 inflammasome.^3^ While this mechanism has not been fully clarified, it has been suggested that colchicine inhibits inflammasome on 2 levels: first by inhibiting P2X_7_ receptor activation and ASC polymerization, thereby inhibiting the interaction between pyrin-like domains^33^ and, second, by suppressing mitochondrial transport and subsequent approximation of ASC to NLRP3.^34^ Our study recruited patients after hospitalization but before any ventilation support; therefore, the observed clinical benefit of colchicine may be generalizable only within this patient population. The possible consequences of initiating colchicine outpatients with COVID-19 is investigated elsewhere.^4^ Colchicine initiation after mechanical ventilation may be futile and should be studied separately.

### Limitations

This study has limitations. This was an open-label study. It was decided that the use of a placebo and masking of patients and their clinical caregivers would complicate their treatment, which was already fraught with extreme difficulty, as well as delay study initiation and participant recruitment. However, the clinical events that met the definition of the primary clinical end point of the study were quite clearly defined, considering that the need for mechanical ventilation or death are rather hard clinical end points. Many other laboratory parameters could have been evaluated in this study, including interleukin or tumor-necrosis factor levels, which could elucidate the effect of colchicine on various inflammatory pathways. This could not be realized because of logistical constraints. Furthermore, the most important limitation is probably the fact that, because of the relatively small number of clinical events, the statistical robustness of the results is limited, even though the arithmetic difference between the 2 groups was striking. In addition, the study was not powered to detect differences in rare adverse events.

## Conclusions

In this randomized clinical trial, participants who received colchicine had statistically significant improved time to clinical deterioration compared with a control group that did not receive colchicine. However, the observed difference was based on a narrow margin of clinical significance; therefore, these observations should be considered hypothesis generating. There were no differences in hs cTn or C-reactive protein levels between the groups.

## Notes

Supplement 1. **Trial Protocol**

Supplement 2. **eFigure. **Levels of High-Sensitivity Troponin, D-Dimer, and C-Reactive Protein in the 2 Groups **eTable. **Laboratory Evaluations at Baseline Plus Maximum or Minimum Values

Supplement 3. **Data Sharing Statement**
