# Supplementary material for: Effect of Colchicine vs Standard Care on Cardiac and Inflammatory Biomarkers and Clinical Outcomes in Patients Hospitalized With Coronavirus Disease 2019: The GRECCO-19 Randomized Clinical Trial
Source: JAMA Netw Open. 2020 Jun 24;3(6):e2013136. doi: 10.1001/jamanetworkopen.2020.13136 (PMC7315286; doi:10.1001/jamanetworkopen.2020.13136)
Supplement: Supplement 1. — Trial Protocol [file jamanetwopen-3-e2013136-s001.pdf]

CLINICAL TRIAL PROTOCOL AMENDMENT

Clinical Trial Title:

ADMINISTRATION OF COLCHICINE IN PATIENTS WITH CORONAVIRUS DISEASE-19 (COVID-19) FOR THE PREVENTION OF DISEASE PROGRESSION AND COMPLICATIONS – THE GRECCO-19 STUDY

(The GReek study in the Effects of Colchicine in Covid-19 cOmplications prevention)

Amendment Document Status:	Final
Amendment Version:	1.0
Date:	08 April 2020
Clinical Trial Phase:	II
Clinical Trial Code:	GRECCO-19
EUDRACT:	2020-001455-40
Sponsor:	Hellenic Society of Rhythmology
Clinical Research Organisation:	Zeincro Hellas S.A.<br>30 Anapafseos St.,<br>Vrilissia 152 35<br>Athens<br>Tel: +30 210 8047709<br>Fax: +30 210 6134695<br>URL: www.zeincro.com

HELLENIC SOCIETY OF RYTHMOLOGY

Confidential
This protocol contains confidential information belonging to the Sponsor. Except as may be otherwise agreed to in writing, by accepting or reviewing these materials you agree to keep such information in confidence and not to disclose it to others (except where required by applicable legislation) nor use it for unauthorized purposes. In the event of suspected or actual breach of this obligation, the Sponsor should be promptly notified.

1. SIGNATURE PAGE

1.1. Sponsor's Representative Signature

Signature	Date

1.2. Protocol Authors' Signatures

Signature<br>*Spyridon Defteraios*	Date

Signature<br>*Dimitrios Vrachatis*	Date

Signature<br>*Georgios Giannopoulos*	Date

1.3. Investigator's Signature

Clinical Trial Protocol Amendment:

The Principal Investigator agrees to the conduct of the clinical trial, as described in this protocol amendment, in accordance with the national/regional regulations and guidance based on ICH/GCP guidelines on good clinical practice and guidance on the conduct of clinical trials during the COVID-19 pandemic and the Declaration of Helsinki. Any protocol amendment must be agreed upon by the Investigator and the Sponsor and documented in writing. By written agreement to this protocol amendment, the Investigator agrees to allow direct access to all documents, including source documents, to authorised people representing the Sponsor (including clinical trial monitors and auditors) and to the regulatory authorities.

Signature **Date**

Full Name

2. CONTACT INFORMATION OF DESIGNATED PERSONNEL

Safety Manager:
Pharmacovigilance and Safety Department, Zeincro
Telephone: *+30 210 80 47 709*
Fax: *+30 210 8047 634*
Email: *dangelidis@zeincro.com*, *safety@zeincro.com*
Address: *30 Anapafseos Street, 152 35, Vrilissia, Athens, Greece*

3. PREVIOUS PROTOCOL AMENDMENTS

There are no previous amendments.

4. IMPLEMENTATION OF CURRENT PROTOCOL AMENDMENT

Amendment Number	Date	Site in which it is subject to implementation
1	08 April 2020	All sites

TABLE OF CONTENTS

1. SIGNATURE PAGE 2
1.1. Sponsor's Representative Signature 2
1.2. Protocol Authors' Signatures 2
1.3. Investigator's Signature 3
2. CONTACT INFORMATION OF DESIGNATED PERSONNEL 3
3. PREVIOUS PROTOCOL AMENDMENTS 4
4. IMPLEMENTATION OF CURRENT PROTOCOL AMENDMENT 4
5. TABLE OF ABBREVIATIONS AND TERMS 8
6. SUMMARY OF AMENDMENTS 9
7. PARTS OF THE PROTOCOL AMENDED 9
7.1. Section 4 "Synopsis", pages 8-9 9
7.2. Section 4 "Synopsis", page 9 10
7.3. Section 7 "Population", page 12 10
7.4. Section 8 "Exclusion criteria", pages 12-13 11
7.5. Section 9 "Design and schedule", page 16 12
7.6. Section 11 "Procedures at each visit", page 17 12
7.7. Section 12 "Medication", page 18 13
7.8. Section 12 "Medication", page 18 14
7.9. Section 13.1 "Adverse Event Definition", Page 19 16
8. SYNOPSIS 17
9. MEDICINAL PRODUCT AND BACKGROUND 20
10. RESEARCH HYPOTHESIS 21
11. POPULATION 21
12. ELIGIBILITY CRITERIA 22
12.1. Inclusion criteria 22
12.2. Exclusion criteria 22
12.3. Definition of enrolled in the clinical trial 23
12.4. Withdrawal of participants from the treatment or assessment 23
12.5. Withdrawal procedure 23
12.6. Early termination of the clinical trial 24
12.7. Replacement of sites 24
12.8. Replacements of participants 24
12.9. Previous and concomitant treatment 24

13. DESIGN AND SCHEDULE 24
14. ENDPOINTS 25
14.1. Biochemical phase 25
14.2. Clinical phase 26
15. PROCEDURES AT EACH VISIT 26
16. MEDICATION 27
16.1. Clinical trial treatment 27
16.2. Other treatments 28
16.3. Special conditions 29
17. SAFETY ASSESSMENT 29
17.1. Adverse Event Definition 30
17.2. Definition of an Adverse Drug Reaction 31
17.3. Reporting Requirements for Severity and Seriousness 31
17.4. Causality Assessment 32
17.5. Adverse Events Reporting 32
17.5.1. Immediate reporting requirements from Investigator to Sponsor 32
17.5.2. Other reporting requirements 33
17.5.3. Pregnancy reporting 33
17.5.4. Congenital Anomalies / Birth Defects 34
17.6. Adverse Event Reporting Period 34
17.7. Follow-up 34
18. STATISTICAL ANALYSIS 35
18.1. Analysis population 35
18.2. Statistical analysis methodology 35
18.3. Exploratory analyses 35
18.4. Required Sample Size 35
19. DATA HANDLING AND STUDY MONITORING 36
19.1. Monitoring, data collection and clinical trial management 36
19.2. Quality Assurance 36
20. REGULATORY AND ETHICS CONSIDERATIONS 37
20.1. Regulatory approvals 37
20.2. Ethical conduct of the clinical trial 37
21. STUDY MANAGEMENT CONSIDERATIONS 37
21.1. Labelling 38
21.2. Storage 38
21.3. Destruction of investigational products 38

21.4. Use of information and publications 38
21.5. Confidentiality and protection of personal data 38
21.6. Medical records and storage and maintenance of records 39
21.7. Training of clinical trial site personnel 40
21.8. Protocol and ICF amendments 41
21.9. Early discontinuation of the clinical trial 41
21.10. Critical documentation 41
21.11. Audits and inspections 41
21.12. Participant Information and Consent 42
21.13. Completion of the clinical trial 43
21.14. Participants' Insurance 43
22. LIMITATIONS OF RESEARCH METHODS 43
23. REFERENCES 44

LIST OF TABLES

Table 1: Semi-quantititative clinical grading scale 26
Table 2: AE severity grading scale 32

5. TABLE OF ABBREVIATIONS AND TERMS

Abbreviation	Term
ALT	Alanine aminotransferase
AST	Aspartate aminotransferase
BMI	Body-mass index
CRF	Case Report Form
CRO	Clinical Research Organisation
eCRF	Electronic Case Report Form
CRP	C-reactive protein
CPK	Creatine phosphokinase
CTCAE	Common Terminology Criteria for Adverse Events
eGFR	Estimated glomerular filtration rate
GCP	Good Clinical Practice
GMP	Good Manufacturing Practice
HbA1c	Glycosylated haemoglobin
hs-cTn	High-sensitivity cardiac troponin
ICF	Informed Consent Form
ICH	International Conference on Harmonisation
NCI	National Cancer Institute of the United States of America
PaO2	Arterial blood partial pressure of oxygen
RT PCR	Reverse transcription polymerase chain reaction
UNL	Upper normal limit
AE	Adverse event
ECG	Electrocardiogram
SAE	Serious adverse event

6. SUMMARY OF AMENDMENTS

Due to practical difficulties in the implementation of all study assessments, owing to the extraordinary workload of employees in the healthcare sector throughout Greece and in an effort to save healthcare resources, a reduction in study visits and only optional recording of some investigations has been decided. Exclusion criteria and instructions for use of colchicine in special population groups were amended so as to include all the information from the summary of characteristics of all available colchicine products in the Greek market, thus not leaving any doubt for its prescribing to the participants.

7. PARTS OF THE PROTOCOL AMENDED

This protocol amendment includes all the following changes:

7.1. Section 4 "Synopsis", pages 8-9

Change from:

"Exclusion criteria:

1. Pregnancy, breastfeeding or unwillingness to take effective contraceptive measures during the clinical trial in women and men of childbearing potential.
2. Known hypersensitivity to colchicine or ~~contraindication to its use~~.
3. Severe hepatic impairment.
4. Estimated glomerular filtration rate (eGFR) <20 ml/min/1.73m^2.
5. Treating physician's clinical judgement that the patient will require mechanical respiratory support within 24 hours.
6. Any condition or circumstances which, at the discretion of the treating physician, would prevent the indicated follow-up of the participant.
7. Electrocardiographic QTc interval>450 msec.
8. Participation in a clinical trial with an investigational product (drug or medical device) or intervention.
9. Under treatment with colchicine for other indications.
10. A subject that, at the discretion of the Investigator, is unable to comply with the requirements of the clinical trial or his/her participation in it may put him/her at unacceptable risks for his/her health."

Change to:

"Exclusion criteria:

If any of the following criteria is met, the subject cannot participate in the clinical trial:

1. Pregnancy, breastfeeding or unwillingness to take effective contraceptive measures during the clinical trial in women and men of childbearing potential.
2. Known hypersensitivity to colchicine **or to any of the excipients of the product (lactose, gum arabic, sucrose, magnesium stearate, microcrystalline cellulose, polyvinylpyrrolidone, methylene casein, erythrosine lacquer).**
3. Severe hepatic impairment.
4. Estimated glomerular filtration rate (eGFR) <20 ml/min/1.73m^2.

5. Treating physician's clinical judgement that the patient will require mechanical respiratory support within 24 hours.
6. Any condition or circumstances which, at the discretion of the treating physician, would prevent the indicated follow-up of the participant.
7. Electrocardiographic QTc interval>450 msec.
8. Participation in a clinical trial with an investigational product (drug or medical device) or intervention.
9. Under treatment with colchicine for other indications.
10. A subject that, at the discretion of the Investigator, is unable to comply with the requirements of the clinical trial or his/her participation in it may put him/her at unacceptable risks for his/her health."
11. **A subject undergoing haemodialysis.**
12. **Severe gastrointestinal failure, severe gastrointestinal disorders, or stomach ulcer.**
13. **Haematological disorders, such as blood diseases.**
14. **Under treatment or had received within the past 14 days drugs belonging to the classes of P-glycoprotein inhibitors or CYP3A4 enzyme inhibitors."**

7.2. Section 4 "Synopsis", page 9

Change from:

"Following the initial visit (Day ~~0~~), data will be recorded at the following timepoints:

- ~~Daily for~~ 10 days for participants in the Biochemical Phase, and if they continue in the Clinical Phase ~~on days 10, 12, 14, 16, 18, 20 and 21~~.
- ~~Over~~ days ~~1 to 10, 12, 14, 16, 18, 20 and 21~~ over participants starting in the Clinical Phase."

Change to:

"Following the initial visit (Day **1**), data will be recorded **every two days** at the following timepoints:

- **Over** 10 days for participants in the Biochemical Phase, and if they continue in the Clinical Phase **over 21 days in total**.
- **For 21** days for participants starting in the Clinical Phase."

7.3. Section 7 "Population", page 12

Change from:

~~"In this clinical trial, men and women over 18 years old, with laboratory confirmed COVID-19 will be enrolled~~ In total, a minimum of 180 participants from sites across Greece will be enrolled in the clinical trial."

Change to:

"In total, a minimum of 180 participants from sites across Greece will be enrolled in the clinical trial."

7.4. Section 8 “Exclusion criteria”, pages 12-13

Change from:

“

1. Pregnancy, breastfeeding or unwillingness to take effective contraceptive measures during the clinical trial in women and men of childbearing potential.
2. Known hypersensitivity to colchicine or ~~contraindication to its use~~.
3. Severe hepatic impairment.
4. Estimated glomerular filtration rate (eGFR) <20 ml/min/1.73m^2.
5. Treating physician’s clinical judgement that the patient will require mechanical respiratory support within 24 hours.
6. Any condition or circumstances which, at the discretion of the treating physician, would prevent the indicated follow-up of the participant.
7. Electrocardiographic QTc interval>450 msec.
8. Participation in a clinical trial with an investigational product (drug or medical device) or intervention.
9. Under treatment with colchicine for other indications.
10. A subject that, at the discretion of the Investigator, is unable to comply with the requirements of the clinical trial or his/her participation in it may put him/her at unacceptable risks for his/her health.”

Change to:

“

1. Pregnancy, breastfeeding or unwillingness to take effective contraceptive measures during the clinical trial in women and men of childbearing potential.
2. Known hypersensitivity to colchicine **or to any of the excipients of the product (lactose, gum arabic, sucrose, magnesium stearate, microcrystalline cellulose, polyvinylpyrrolidone, methylene casein, erythrosine lacquer).**
3. Severe hepatic impairment.
4. Estimated glomerular filtration rate (eGFR) <20 ml/min/1.73m^2.
5. Treating physician’s clinical judgement that the patient will require mechanical respiratory support within 24 hours.
6. Any condition or circumstances which, at the discretion of the treating physician, would prevent the indicated follow-up of the participant.
7. Electrocardiographic QTc interval>450 msec.
8. Participation in a clinical trial with an investigational product (drug or medical device) or intervention.
9. Under treatment with colchicine for other indications.
10. A subject that, at the discretion of the Investigator, is unable to comply with the requirements of the clinical trial or his/her participation in it may put him/her at unacceptable risks for his/her health.”
11. **A subject undergoing haemodialysis.**
12. **Severe gastrointestinal failure, severe gastrointestinal disorders, or stomach ulcer.**
13. **Haematological disorders, such as blood diseases.**
14. **Under treatment or had received within the past 14 days drugs belonging to the classes of P-glycoprotein inhibitors or CYP3A4 enzyme inhibitors.”**

7.5. Section 9 "Design and schedule", page 16

Change from:

"Following the initial visit (Day ~~0~~), data will be recorded at the following timepoints:

- ~~Daily for~~ 10 days for participants in the Biochemical Phase, and if they continue in the Clinical Phase ~~on days 10, 12, 14, 16, 18, 20 and 21~~.
- ~~Over~~ days ~~1 to 10, 12, 14, 16, 18, 20 and 21~~ over participants starting in the Clinical Phase."

Change to:

"Following the initial visit (Day **1**), data will be recorded **every two days** at the following timepoints:

- **Over** 10 days for participants in the Biochemical Phase, and if they continue in the Clinical Phase **over 21 days in total**.
- **For 21** days for participants starting in the Clinical Phase."

7.6. Section 11 "Procedures at each visit", page 17

Change from:

"At the initial visit, the following data will be recorded in the CRF:

1. Demographic data (age, sex, nationality)
2. Full medical history (including comorbidities, concomitant medications, smoking).
3. Full physical examination (including body-mass index [BMI]).
4. ICF signature date.
5. Date of onset of symptoms and positive RT PCR test.
6. Type and posology of treatment for COVID-19.
7. Glycosylated haemoglobin (HbA1c).

At each visit, including the initial one, the following data will be recorded:

1. Any change in the medical history or treatment
2. Physical examination results
3. Adverse events
4. Maximum daily body temperature
5. ~~Electrocardiogram (~~ECG~~)~~
6. hs-cTn
7. Procalcitonin
8. Glucose
9. C-reactive protein (CRP)
10. D-Dimmers
11. Complete Blood Count
12. Blood serum electrolytes (sodium, potassium)
13. Liver function markers (aspartate aminotransferase [AST], alanine aminotransferase [ALT])
14. Kidney function markers (urea, creatinine, eGFR)
15. Creatine phosphokinase (CPK)

16. Lactate dehydrogenase (LDH)
17. Ferritin
18. SARS-CoV-19 viral load ~~mandatory on the 1st and 7th day, with optional measurements at the remaining visits.~~
19. ~~PaO_2 by oximetry twice daily.”~~

Change to:

“At the initial visit, the following data will be recorded in the CRF:

1. Demographic data (age, sex, nationality)
2. Full medical history (including comorbidities, concomitant medications, smoking).
3. Full physical examination (including body-mass index [BMI]).
4. ICF signature date.
5. Date of onset of symptoms and positive RT PCR test.
6. Type and posology of treatment for COVID-19.
7. Glycosylated haemoglobin (HbA1c).
8. **Electrocardiogram (ECG).**
9. **Arterial blood gases, if required to determine eligibility for participation, according to the exclusion criteria.**

At each visit, including the initial one, the following data will be recorded:

1. Any change in the medical history or treatment
2. Physical examination results
3. Adverse events
4. Maximum daily body temperature
5. ECG **upon clinical indications**
6. hs-cTn
7. Procalcitonin
8. Glucose
9. C-reactive protein (CRP)
10. D-Dimmers
11. Complete Blood Count
12. Blood serum electrolytes (sodium, potassium)
13. Liver function markers (aspartate aminotransferase [AST], alanine aminotransferase [ALT])
14. Kidney function markers (urea, creatinine, eGFR)
15. Creatine phosphokinase (CPK)
16. Lactate dehydrogenase (LDH)
17. Ferritin
18. SARS-CoV-19 viral load **optionally, based on clinical utility at the discretion of the treating physicians.**
19. **Arterial blood gases optionally from one measurement, based on clinical indications.”**

7.7. Section 12 “Medication”, page 18

Change from:

"12. MEDICATION

Group A:"

Change to:

"16. MEDICATION

16.1 Clinical trial treatment

Group A:"

7.8. Section 12 "Medication", page 18

Change from:

"Investigators will administer colchicine according to the restrictions and instructions included in the approved Summary of Product Characteristics.

13. SAFETY ASSESSMENT"

Change to:

"Investigators will administer colchicine according to the restrictions and instructions included in the approved Summary of Product Characteristics.

16.2 Other treatments

Monitoring should be increased, when colchicine is combined with active substances that are metabolized by, or interact with, the cytochrome P450 system, particularly with isoenzyme CYP3A4 or P-glycoprotein. Indicatively, such substances are:

- Anti-infective agents: Colchicine toxicity increases when combining treatment with clarithromycin, erythromycin, telithromycin, CYP3A4 substrates and inhibitors, particularly in subjects with pre-existing renal disorders. Other CYP3A4 inhibitors, such as itraconazole, ketoconazole, indinavir, nelfinavir, ritonavir and saquinavir, may increase colchicine toxicity.

- Calcium channel blockers: verapamil and diltiazem.

- Cyclosporine: Colchicine must be used with caution in combination with cyclosporine, due to the possible risk of increased nephrotoxicity and myotoxicity.

- Vitamins: Vitamin B12 absorption may be affected by chronic administration or high doses of colchicine. Vitamin requirements may be increased.

- Concomitant use of atorvastatin, simvastatin, pravastatin, fluvastatin, gemfibrozil, fenofibrate, fenofibric acid or bezafibrate (which are associated with myotoxicity) may promote the appearance of myopathies. Once treatment with colchicine is discontinued, the symptoms usually resolve within one week to several months.

- Treatment with colchicine must not be combined with grapefruit juice intake (a CYP3A4 inhibitor), as colchicine toxicity may be enhanced.

- Antiarrhythmics: amiodarone, dronedarone, disopyramide, quinidine.

- Antivirals such as ritonavir, atazanavir or indinavir (antiviral drugs used to treat an HIV infection).

- Ticagrelor.

- Coumarin anticoagulants, such as warfarin.

- Phenytoin, valproic acid, carbamazepine, phenobarbital.

- Astemizole, terfenadine, methylprednisolone.

- Rifabutin, rifapentine.

- Theophylline.

- Alprazolam, midazolam, triazolam.

- Carbamazepine, pimozide.

- Cisapride.

- Tacrolimus.

- Sildenafil.

- Cilostazol.

- Omeprazole.

- Vinblastine.

16.3 Special conditions

Special warnings and precautions for the use of colchicine must be taken into consideration:

- In the event of diarrhoea, treatment must be discontinued or dose must be reduced.

- Treatment with colchicine in the elderly and debilitated subjects or those who abuse alcohol, must be closely monitored, due to the higher risk of cumulative toxicity in these populations.

- Leukopenia, granulocytopenia, thrombocytopenia, pancytopenia, aplastic anaemia and myelosuppression have been associated with the use of colchicine at therapeutic doses, thus possible AEs must be closely monitored.

- A dose adjustment may be required in subjects with hepatobiliary and renal impairment. These subjects must be closely monitored during treatment. No dose adjustment is required in subjects with mild renal impairment (creatinine clearance 50-80 ml/min), although close monitoring is recommended, due to the possible occurrence of adverse effects. Should these occur, a dose reduction may be necessary. The dose may need to be reduced by half and/or the interval between doses may need to be increased in subjects with moderate renal impairment (creatinine clearance 30-50 ml/min). The use of colchicine is contraindicated in subjects with severe renal impairment (creatinine clearance <30 ml/min). No dose adjustment is required in subjects with mild or moderate hepatic impairment,

although close monitoring is recommended, due to the possible occurrence of adverse effects. Should these occur, a dose reduction may be necessary. The use of colchicine is contraindicated in subjects with severe hepatic impairment.

- Colchicine clearance is reduced in subjects with renal dysfunction.

- Colchicine clearance may be significantly reduced and its plasma half-life may be increased in subjects with hepatic insufficiency.

- Neuromuscular toxicity and rhabdomyolysis have been reported with chronic treatment at therapeutic doses. This risk may be increased in subjects with renal failure and in the elderly (even those with no hepatic or renal failure).

17. SAFETY ASSESSMENT"

7.9. Section 13.1 "Adverse Event Definition", Page 19

Change from:

"Events that are not clearly consistent with the expected pattern of progression of the underlying disease should ~~not~~ be recorded as AEs.

Change to:

"Events that are not clearly consistent with the expected pattern of progression of the underlying disease should be recorded as AEs.

8. SYNOPSIS

**Sponsor's Name:**	Hellenic Society of Rhythmology
**Clinical trial title:**	ADMINISTRATION OF COLCHICINE IN PATIENTS WITH CORONAVIRUS DISEASE-19 (COVID-19) FOR THE PREVENTION OF DISEASE PROGRESSION AND COMPLICATIONS – THE GRECCO-19 STUDY<br>(The GReek study in the Effects of Colchicine in Covid-19 cOmplications prevention)
**Clinical trial code:**	GRECCO-19
**Planned duration of the clinical trial:**	6-month clinical trial duration with 21 observation days for all participants
**Clinical phase:**	II
**Endpoints:**	**Biochemical phase**<br>Primary endpoints:<br>1) Maximum value of high-sensitivity cardiac troponin (hs-cTn) within 10 days from treatment initiation.<br>2) Time for maximum value of C-reactive protein (CRP) to reach levels > 3 x upper normal limit (UNL).<br>Secondary endpoint:<br>1) The number, the type, the severity and the seriousness of total AEs and treatment-related AEs at the end of follow-up.<br>**Clinical phase**<br>Primary endpoint:<br>1) Time to deterioration by 2 points on the clinical status scale, as suggested by the WHO R&D Blueprint committee (32–34). (Table 1).<br>Secondary endpoints:<br>2) Percentage of participants requiring mechanical ventilation at each visit.<br>3) All-cause mortality at the end of follow-up.<br>4) The number, the type, the severity and the seriousness of total AEs and treatment-related AEs at the end of follow-up.<br>Exploratory endpoint:<br>5) Lung function tests in the cured patients.
**Planned number of participants:**	180 in total (60 in the Biochemical Phase with the potential to continue in the Clinical Phase. 180 in total in the Clinical Phase, including participants continuing from the Biochemical Phase).

**Diagnosis and eligibility criteria:**	<u>Inclusion criteria:</u><br><br>In order for a subject to be able to enroll in the clinical trial, he/she should meet <u>all</u> of the following inclusion criteria:<br><br>1. Subjects >18 years old with laboratory confirmed SARS-COV-2 [by reverse transcription polymerase chain reaction (RT PCR)], who present with clinical symptoms including body temperature >37.5 ^oC.<br>**<u>AND</u>**<br>2. <u>At least two</u> of the following criteria:<br>1) persistent cough<br>2) persistent throat pain<br>3) anosmia, ageusia<br>4) asthenia<br>5) Arterial blood partial pressure of oxygen (PaO2)<95 mmHg.<br><br><u>Exclusion criteria:</u><br><br>If any of the following criteria is met, the subject cannot participate in the clinical trial:<br><br>1. Pregnancy, breastfeeding or unwillingness to take effective contraceptive measures during the clinical trial in women and men of childbearing potential.<br>2. Known hypersensitivity to colchicine or to any of the excipients of the product (lactose, gum arabic, sucrose, magnesium stearate, microcrystalline cellulose, polyvinylpyrrolidone, methylene casein, erythrosine lacquer).<br>3. Severe hepatic impairment.<br>4. Estimated glomerular filtration rate (eGFR) <20 ml/min/1.73m^2.<br>5. Treating physician's clinical judgement that the patient will require mechanical respiratory support within 24 hours.<br>6. Any condition or circumstances which, at the discretion of the treating physician, would prevent the indicated follow-up of the participant.<br>7. Electrocardiographic QTc interval>450 msec.<br>8. Participation in a clinical trial with an investigational product (drug or medical device) or intervention.<br>9. Under treatment with colchicine for other indications.<br>10. A subject that, at the discretion of the Investigator, is unable to comply with the requirements of the clinical trial or his/her participation in it may put him/her at unacceptable risks for his/her health."<br>11. A subject undergoing haemodialysis.<br>12. Severe gastrointestinal failure, severe gastrointestinal disorders, or stomach ulcer.<br>13. Haematological disorders, such as blood diseases.

	14. Under treatment or had received within the past 14 days drugs belonging to the classes of P-glycoprotein inhibitors or CYP3A4 enzyme inhibitors.
**Methodology:**	Participants will be randomized in two groups, at a 1:1 ratio.<br>Group A: participants of group A will be receiving the optimal treatment based on the algorithm of the Expert Committee of the Ministry of Health.<br>Group B: participants of group B will be additionally receiving colchicine.<br>Following the initial visit (Day 1), data will be recorded every two days at the following timepoints:<br>➢ Over 10 days for participants in the Biochemical Phase, and if they continue in the Clinical Phase over 21 days in total.<br>➢ For 21 days for participants starting in the Clinical Phase.
**Safety:**	The number, the type, the severity and the seriousness of total AEs and treatment-related AEs at the end of follow-up will be collected during the entire follow-up.
**Statistical methods:**	Analysis of primary objectives will be performed independently upon completion of all necessary assessments required for them. Analysis of secondary and exploratory objectives will be performed after clinical trial completion.<br>Quantitative (continuous) variables will be summarized using descriptive statistics (mean value, standard deviation, median and range). Qualitative (categorical) variables will be presented as frequencies and percentages (N, %). Any differences between the mean values of the continuous variables will be assessed using parametric statistical tests (e.g. t-test, ANOVA) or non-parametric equivalents (e.g. Wilcoxon signed-rank test, Mann-Whitney Kruskal-Wallis).<br>All statistical tests will be 2-sided and will be conducted at a significance level α=5%. The exact p values will be reported, even for non-significant results. All analyses will be conducted using a validated statistical analysis programme (e.g. IBM-SPSS 21 and/or R 3.3.2).<br>Adverse event and serious adverse event coding will be performed by Preferred Term and System Organ Class, based on the current version of MedDRa medical dictionary. Medications will be coded using the current MedDRA dictionary and the World Health Organization Drug Dictionary (WHO-DD).
**Version:**	Final 1.0
**Date:**	08 April 2020

9. MEDICINAL PRODUCT AND BACKGROUND

Colchicine is a drug administered for years in cardiology. It is classically described as a treatment for acute pericarditis (1). In fact, in the recent European Society of Cardiology guidelines for the management of pericardial diseases it is suggested that it may be used for the prevention of post-pericardiotomy syndrome (1). Moreover, in a recent expert consensus statement it is suggested that colchicine may be used for the prevention of atrial fibrillation recurrences following cardiac surgery or following radiofrequency ablation procedures (2). Beyond these indications, colchicine has been used safely in research in a number of different clinical scenarios, including acute myocardial infarction, giving promising results (3, 4)

As far as patients with COVID-19 are concerned, it seems from the first reports that the myocardium is quite frequently involved (published data describe up to 20% of cases, whereas anecdotal reports are even suggesting 50-60% of patients). In fact, in a recent clinical trial from the initial epicentre of the epidemic in Wuhan in 191 patients, the elevation of myocardial necrosis enzymes is associated as a negative independent prognostic factor of patients' need for mechanical respiratory support (5). In another clinical trial from Wuhan among 150 people with COVID-19, 7% of deaths was attributed to myocarditis with circulatory failure, while in 33% myocarditis had played a crucial role in the final adverse clinical outcome (6).

In addition, people suffering from COVID-19 often develop acute respiratory distress syndrome and acute lung injury (ARDS/ALI). The uncontrolled progressive pulmonary inflammation causes acute diffuse alveolar damage resulting in pulmonary infiltrates and, clinically, acute respiratory failure. SARS-CoV-2 infection is associated with direct and indirect cardiovascular complications. Currently available studies indicate that patients with a pre-existing cardiovascular disease may be at a high risk of SARS-CoV-2 infection. Furthermore, patients with COVID-19 and a pre-existing cardiovascular disease are at an increased risk of adverse outcomes. Published reports from cardiac complications of SARS-CoV-2 infection include arrythmia, acute heart disease, acute onset heart failure, myocardial infarction, myocarditis, and cardiac arrest. It has been also reported that patients suffering from COVID-19 present with elevation of myocardial cardiac necrosis enzymes, which is an adverse prognostic factor (5).

In experimental models it has been shown that NLRP3 inflammasome is a key mediator in ARDS/ALI (7–10), while *in silico* models have shown that in the new SARS-CoV-2, proteins such as viroporins E, 3a and 8A play a crucial role in viral replication and its pathogenesis (11). Additionally, it has been shown that these three proteins promote the activation of NLRP3 inflammasome (12–16).

Besides, cell entry of SARS-CoV-2 depends on binding of the viral S proteins to cellular receptors and on viral protein priming by host cell proteases (17, 18). Therefore, factors that may affect clathrin-mediated endocytosis [a process that is, in part, regulated by microtubule remodelling (19)] could potentially decelerate viral infection of cells (20).

Colchicine is a lipid-soluble alkaloid (21), which after oral administration is absorbed from the jejunal and ileal mucosa. Peak plasma concentration is reached 1-2 hours following a single oral dose, while its maximal anti-inflammatory effect develops over 24 to 48 hours. This time is necessary for the drug to accumulate into granulocytes and monocytes, reaching concentrations several times higher than its peak plasma concentration (22). There, it can be detected for days after the last administration (23).

Colchicine exerts its action through binding to unpolymerized tubulin heterodimers. The formation of this stable complex substantially inhibits any process that requires cytoskeletal changes and in which microtubules participate (24).

Moreover, colchicine is a non-selective inhibitor of NLRP3 inflammasome (25). While initially thought of merely as an inhibitor of microtubule polymerization and leucocyte infiltration, a significant part of colchicine anti-inflammatory action seems to be attributed to the inhibition of the NLRP3 inflammasome (26). Colchicine inhibits inflammasome's action on two levels: it inhibits P2X7 receptor activation and ASC (Apoptosis-associated speck-like protein containing a CARD), polymerization, thereby inhibiting the interaction between pyrin-like domains (27). It has also been found to suppress the transport of mitochondria and the subsequent approximation of ASC to NLRP3, indicating that microtubules create the sites for the interaction of different parts of the inflammasome, and which eventually leads to its activation (28). Colchicine has been shown to limit IL-1β production as a response to various NLRP3 inflammasome inducers in a dose-dependent fashion. For example, administration of colchicine in patients with acute coronary syndrome effectively suppressed local production of interleukins IL-1β, IL-18 and IL-6, an effect that was attributed to inhibition of inflammasome's action (29, 30).

Colchicine has been safely administered to patients with acute myocardial infarction, both in the acute phase and in the post-myocardial infarction period (4, 31).

10. RESEARCH HYPOTHESIS

Based on the foregoing data, the question arises as to whether colchicine administered at a relatively low dose could, mainly through the inhibition of the inflammasome and, possibly, due to the processes of SARS-CoV-2 endocytosis into the endothelial respiratory cells, have an effect to a clinically significant extent on the clinical laboratory outcome of patients with COVID-19 as well as on the prevention of myopericarditis.

The protocol will have two phases: the "Biochemical" phase, during which, differences in biomarkers between the two groups will be assessed, and the "Clinical" phase, during which, differences in clinical endpoints will be assessed.

The efficacy and safety of the administration of colchicine in cardiology is well-established and recommended in guidelines. The increasing experience in the management of patients with SARS-CoV-2 infection shows that the virus causes direct and indirect cardiological complications with adverse consequences for the patients both in the short-term and in the long-term. These data have rendered promising the administration of colchicine in combination with the recommended treatment to COVID-19 patients within the context of phase II and III clinical trials abroad in outpatients and inpatients. In this trial, administration of colchicine will be performed to subjects at an increased risk of cardiological complications under strict medical supervision. Therefore, the benefit from the administration of colchicine outweighs its potential risks.

11. POPULATION

In total, a minimum of 180 participants from sites across Greece will be enrolled in the clinical trial. This trial will include 2 phases. In the Biochemical Phase, approximately 60 participants, who may also be subsequently assessed in the Clinical phase, will be enrolled. In the Clinical Phase, a minimum of 180 participants will be enrolled.

12. ELIGIBILITY CRITERIA

12.1. Inclusion criteria

In order for a subject to be able to enroll in the clinical trial, he/she should meet all of the following inclusion criteria:

1. Subjects >18 years old with laboratory confirmed SARS-COV-2 [by reverse transcription polymerase chain reaction (RT PCR)], who present with clinical symptoms including body temperature >37.5 ^{o}C.
   AND
2. At least two of the following criteria:
   6) persistent cough
   7) persistent throat pain
   8) anosmia, ageusia
   9) asthenia
   10) Arterial blood partial pressure of oxygen (PaO2)<95 mmHg.

12.2. Exclusion criteria

If any of the following criteria is met, the subject cannot participate in the clinical trial:

1. Pregnancy, breastfeeding or unwillingness to take effective contraceptive measures during the clinical trial in women and men of childbearing potential.
2. Known hypersensitivity to colchicine or to any of the excipients of the product (lactose, gum arabic, sucrose, magnesium stearate, microcrystalline cellulose, polyvinylpyrrolidone, methylene casein, erythrosine lacquer).
3. Severe hepatic impairment.
4. Estimated glomerular filtration rate (eGFR) <20 ml/min/1.73m^2.
5. Treating physician's clinical judgement that the patient will require mechanical respiratory support within 24 hours.
6. Any condition or circumstances which, at the discretion of the treating physician, would prevent the indicated follow-up of the participant.
7. Electrocardiographic QTc interval>450 msec.
8. Participation in a clinical trial with an investigational product (drug or medical device) or intervention.
9. Under treatment with colchicine for other indications.
10. A subject that, at the discretion of the Investigator, is unable to comply with the requirements of the clinical trial or his/her participation in it may put him/her at unacceptable risks for his/her health.
11. A subject undergoing haemodialysis.
12. Severe gastrointestinal failure, severe gastrointestinal disorders, or stomach ulcer.
13. Haematological disorders, such as blood diseases.
14. Under treatment or had received within the past 14 days drugs belonging to the classes of P-glycoprotein inhibitors or CYP3A4 enzyme inhibitors.

12.3. Definition of enrolled in the clinical trial

Enrolled in the clinical trial is defined as the participant who has signed the Informed Consent Form (ICF).

All participants or their legal representatives must sign and date the informed consent form by hand prior to inclusion in the clinical trial. In case a subject is not able to give his/her written consent and wants to participate in the clinical trial, the consent form may be signed in his/her place by an impartial witness.

12.4. Withdrawal of participants from the treatment or assessment

As the decision for the treatment lies to the Treating Physician and he/she is not bound to the participation of the participants in the clinical trial, the Investigator has the right to withdraw a participant from the clinical trial at any time. Additionally, participants have the right to voluntarily drop-out from the clinical trial at any time and for any reason. Reasons for discontinuation of treatment with the medicinal product or withdrawal from the clinical trial may indicatively include the following:

- Revocation of participant's consent at any time.
- The participant meets an exclusion criterion (either recently developed or not previously recognized) that precludes further participation in the clinical trial.
- Enrollment or planned participation in any clinical trial, at any time during the observation period of the clinical trial, in which the participant has been or will be exposed to an investigational product (drug or medical device) or intervention.
- Investigator's decision.
- Major protocol deviation or violation.
- Participant is lost to follow-up.

12.5. Withdrawal procedure

Participants, who voluntarily discontinue the clinical trial treatment or withdraw from the clinical trial, should be asked about the reasons for discontinuation and the occurrence of any adverse events, and, where applicable, must be examined and evaluated by the Investigator in accordance with the procedures defined in the End of Treatment or Early Discontinuation Visit. Adverse events (AEs) must be monitored until their resolution or stabilization.

In addition, if a participant withdraws early from the clinical trial, he/she will be contacted by phone or other methods to assess his/her status at the end of the clinical trial, unless the participant has actively withdrawn his/her consent for all forms of contact.

If the participant specifically withdraws his/her consent for contacting him/her for additional information, it is not possible for clinical trial-related contacts to be conducted, unless it is about issues pertaining to pharmacovigilance. Data that will have been collected until the withdrawal will be included in the clinical trial analyses.

12.6. Early termination of the clinical trial

The sponsor reserves the right to terminate the clinical trial overall or at a specific clinical trial location, at any time for the following reasons:

- Not achieving the expected target enrollment, overall or at specific sites.
- Occurrence of any information on efficacy/safety that could significantly impact clinical trial continuation or any other administrative reasons.
- Violation of clinical trial protocol or any term of the conditions of the clinical trial agreement by a participating site or an Investigator.

Following such a decision, the Investigator must contact all participants immediately, if the decision is due to safety concerns or within 1 week, if the decision is due to other reasons. As defined by the Sponsor, all the clinical trial material should be collected, and all case report forms (CRF) should be completed as fully as possible.

The Sponsor will inform the Investigator if the clinical trial is discontinued or if the Sponsor decides to discontinue the clinical trial.

12.7. Replacement of sites

The Sponsor has the right to replace any site at any time. Reasons for the replacement of a site may indicatively include the following:

- Too low enrollment rate of participants in the clinical trial.
- Insufficient compliance with the protocol.
- Inaccurate or incomplete data recording.
- Non-compliance with the guidelines for Good Clinical Practice (GCP) or any other relevant local legislation or guideline.

12.8. Replacements of participants

In case of a participant’s drop-out, he/she will be replaced so that the planned number of participants per phase is achieved.

12.9. Previous and concomitant treatment

Colchicine requires caution when taken together with P-glycoprotein inhibitors and strong or moderate cytochrome P-450 inhibitors. Caution is also warranted in subjects with hepatic or renal failure.

13. DESIGN AND SCHEDULE

The clinical trial will be conducted with a prospective design, of two parallel groups, with randomization at a 1:1 ratio, and competitive enrollment between sites. Randomization

will be performed via a special form, which will be maintained in confidence at each site. Participants at each site will be randomized in two groups, A and B.

Group A: participants of group A will be receiving the optimal treatment based on the applicable algorithm of the Expert Committee of the Ministry of Health.

Group B: participants of group B will be additionally receiving colchicine.

More instructions on the method of administration of colchicine are included below in section 16 “MEDICATION“.

For the purposes of this study, a randomization scheme will be formed with the use of a random number generator. Permuted block design will be implemented, where random block sizes will be used to avoid predictability.

The clinical trial will be conducted in two phases: the Biochemical Phase and the Clinical Phase. Following completion of enrollment and assessment of the 60 participants of the Biochemical Phase, an interim analysis of the efficacy and safety of the treatment will be performed to decide on clinical trial continuation. All participants completing the follow-up of the Biochemical Phase will continue in the Clinical Phase. 180 participants in total will be enrolled in both phases. Analysis of primary objectives will be performed independently upon completion of all necessary assessments required for them. Analysis of secondary and exploratory objectives will be performed after clinical trial completion.

Given the extremely critical situation due to the COVID-19 pandemic, results will be analysed every 20 participants completing the protocol with the purpose of becoming immediately available to the scientific community in case there are significant findings.

Following the initial visit (Day **1**), data will be recorded **every two days** at the following timepoints:

- **Over** 10 days for participants in the Biochemical Phase, and if they continue in the Clinical Phase **over 21 days in total**.
- **For 21** days for participants starting in the Clinical Phase.

Participants in the need of mechanical ventilation will be early withdrawn from the clinical trial. Data collected for them up to the point of withdrawal will be included in the analyses. The overall clinical trial duration will be 6 months.

14. ENDPOINTS

In each one of the following endpoints the difference between the results of the two participant groups will be analysed.

14.1. Biochemical phase

Primary endpoints:

1) Maximum value of high-sensitivity cardiac troponin (hs-cTn) within 10 days from treatment initiation.
2) Time for maximum value of C-reactive protein (CRP) to reach levels > 3 x upper normal limit (UNL).

Secondary endpoint:

1) The number, the type, the severity and the seriousness of total AEs and treatment-related AEs at the end of follow-up.

14.2. Clinical phase

Primary endpoints:

1) Time to deterioration by 2 points on the clinical status scale, as suggested by the WHO R&D Blueprint committee (32–34). (Table 1).

Table 1: Semi-quantitative clinical grading scale

Grade	Description
1	not hospitalized patient, able to resume normal activities
2	not hospitalized patient, unable to resume normal activities
3	hospitalized patient, not requiring oxygen administration
4	hospitalized patient, requiring oxygen therapy
5	hospitalized patient, requiring high-flow oxygen therapy, non-invasive mechanical ventilation, or both
6	hospitalized patient, requiring ECMO or mechanical ventilation
7	death

Secondary endpoints:

1) Percentage of participants requiring mechanical ventilation at each visit.
2) All-cause mortality at the end of follow-up.
3) The number, the type, the severity and the seriousness of total AEs and treatment-related AEs at the end of follow-up.

Exploratory endpoint:

4) Lung function tests in the cured patients.

15. PROCEDURES AT EACH VISIT

The procedures described per visit concern participants in both the Biochemical and the Clinical Phase.

At the initial visit, the following data will be recorded in the CRF:

10. Demographic data (age, sex, nationality)
11. Full medical history (including comorbidities, concomitant medications, smoking).
12. Full physical examination (including body-mass index [BMI]).
13. ICF signature date.
14. Date of onset of symptoms and positive RT PCR test.
15. Type and posology of treatment for COVID-19.
16. Glycosylated haemoglobin (HbA1c).
17. Electrocardiogram (ECG).
18. Arterial blood gases, if required to determine eligibility for participation, according to the exclusion criteria.

At each visit, including the initial one, the following data will be recorded:

1. Any change in the medical history or treatment
2. Physical examination results
3. Adverse events
4. Maximum daily body temperature
5. ECG upon clinical indications
6. hs-cTn
7. Procalcitonin
8. Glucose
9. C-reactive protein (CRP)
10. D-Dimmers
11. Complete Blood Count
12. Blood serum electrolytes (sodium, potassium)
13. Liver function markers (aspartate aminotransferase [AST], alanine aminotransferase [ALT])
14. Kidney function markers (urea, creatinine, eGFR)
15. Creatine phosphokinase (CPK)
16. Lactate dehydrogenase (LDH)
17. Ferritin
18. SARS-CoV-19 viral load optionally, based on clinical utility at the discretion of the treating physicians.
19. Arterial blood gases optionally from one measurement, based on clinical indications.

Exploratory tests:

In case of cure from SARS-CoV-2, defined as two negative RT PCR tests with an interval of 24-72 hours, spirometry testing of pulmonary function will be performed optionally, within 15 days.

16. MEDICATION

16.1. Clinical trial treatment

Group A:

Participants of group A will be receiving only the defined treatment based on the applicable algorithm of the Expert Committee of the Ministry of Health.

Group B:

Participants of group B will be receiving the defined treatment based on the applicable algorithm of the Expert Committee of the Ministry of Health, and additionally oral colchicine.

Colchicine loading: Colchicine will be initially administered as a single dose of 1.5 mg and one hour later, provided there are no adverse gastrointestinal effects, an additional 0.5 mg.

In case of azithromycin coadministration, the initial loading will be 1 mg in total.

Maintenance dose: Then, participants will be receiving 0.5 mg colchicine twice a day, except for participants with a body weight <60kg who will be receiving 0.5 mg once a day.

Investigators will administer colchicine according to the restrictions and instructions included in the approved Summary of Product Characteristics.

16.2. Other treatments

Monitoring should be increased, when colchicine is combined with active substances that are metabolized by, or interact with, the cytochrome P450 system, particularly with isoenzyme CYP3A4 or P-glycoprotein. Indicatively, such substances are:

- Anti-infective agents: Colchicine toxicity increases when combining treatment with clarithromycin, erythromycin, telithromycin, CYP3A4 substrates and inhibitors, particularly in subjects with pre-existing renal disorders. Other CYP3A4 inhibitors, such as itraconazole, ketoconazole, indinavir, nelfinavir, ritonavir and saquinavir, may increase colchicine toxicity.

- Calcium channel blockers: verapamil and diltiazem.

- Cyclosporine: Colchicine must be used with caution in combination with cyclosporine, due to the possible risk of increased nephrotoxicity and myotoxicity.

- Vitamins: Vitamin B12 absorption may be affected by chronic administration or high doses of colchicine. Vitamin requirements may be increased.

- Concomitant use of atorvastatin, simvastatin, pravastatin, fluvastatin, gemfibrozil, fenofibrate, fenofibric acid or bezafibrate (which are associated with myotoxicity) may promote the appearance of myopathies. Once treatment with colchicine is discontinued, the symptoms usually resolve within one week to several months.

- Treatment with colchicine must not be combined with grapefruit juice intake (a CYP3A4 inhibitor), as colchicine toxicity may be enhanced.

- Antiarrhythmics: amiodarone, dronedarone, disopyramide, quinidine.

- Antivirals such as ritonavir, atazanavir or indinavir (antiviral drugs used to treat an HIV infection).

- Ticagrelor.

- Coumarin anticoagulants, such as warfarin.

- Phenytoin, valproic acid, carbamazepine, phenobarbital.

- Astemizole, terfenadine, methylprednisolone.

- Rifabutin, rifapentine.

- Theophylline.

- Alprazolam, midazolam, triazolam.

- Carbamazepine, pimozide.

- Cisapride.

- Tacrolimus.

- Sildenafil.

- Cilostazol.

- Omeprazole.

- Vinblastine.

16.3. Special conditions

Special warnings and precautions for the use of colchicine must be taken into consideration:

- In the event of diarrhoea, treatment must be discontinued or dose must be reduced.

- Treatment with colchicine in the elderly and debilitated subjects or those who abuse alcohol, must be closely monitored, due to the higher risk of cumulative toxicity in these populations.

- Leukopenia, granulocytopenia, thrombocytopenia, pancytopenia, aplastic anaemia and myelosuppression have been associated with the use of colchicine at therapeutic doses, thus possible AEs must be closely monitored.

- A dose adjustment may be required in subjects with hepatobiliary and renal impairment. These subjects must be closely monitored during treatment. No dose adjustment is required in subjects with mild renal impairment (creatinine clearance 50-80 ml/min), although close monitoring is recommended, due to the possible occurrence of adverse effects. Should these occur, a dose reduction may be necessary. The dose may need to be reduced by half and/or the interval between doses may need to be increased in subjects with moderate renal impairment (creatinine clearance 30-50 ml/min). The use of colchicine is contraindicated in subjects with severe renal impairment (creatinine clearance <30 ml/min). No dose adjustment is required in subjects with mild or moderate hepatic impairment, although close monitoring is recommended, due to the possible occurrence of adverse effects. Should these occur, a dose reduction may be necessary. The use of colchicine is contraindicated in subjects with severe hepatic impairment.

- Colchicine clearance is reduced in subjects with renal dysfunction.

- Colchicine clearance may be significantly reduced and its plasma half-life may be increased in subjects with hepatic impairment.

- Neuromuscular toxicity and rhabdomyolysis have been reported with chronic treatment at therapeutic doses. This risk may be increased in subjects with renal failure and in the elderly (even those with no hepatic or renal failure).

17. SAFETY ASSESSMENT

The following parameters will be actively assessed during the entire participation in this clinical trial:

- The number, the seriousness, the severity and the type of adverse events (AEs).
- The causal or non-causal association of AEs with treatment.
- Th date of onset and date of resolution or status of the AE during the last clinical trial assessment.

17.1. Adverse Event Definition

An AE is defined as the appearance or worsening of an unintended sign, symptom or medical condition occurring after signing the ICF, even if this event is not considered to be related to the administration of the clinical trial treatment. Medical conditions/diseases present at clinical trial initiation are only considered AEs if they worsen after signing the ICF. The decision as to whether examination findings should be classified as AEs is up to medical judgement.

An AE can therefore be, but not limited to, any of the following:

- Any unfavourable and unintended sign (including any abnormal laboratory finding), symptom, or disease temporally associated with the use of a medicinal product, whether or not considered related to the medicinal product.
- An exacerbation of an existing disease.
- Recurrence of an intermittent medical condition not present at baseline.
- Any deterioration in a laboratory value or other physical examination (if judged by the Investigator to have unexpectedly worsened in severity or frequency or changed in nature at any time during the clinical trial) that is associated with symptoms or leads to a change in clinical trial treatment or concomitant treatment or clinical trial drug discontinuation.
- Overdose, abuse, off-label use, misuse and medication error associated with a medicinal product.
- Lack of therapeutic efficacy of a medicinal product.

A pre-existing medical condition should be recorded as an AE only if the frequency, severity, or character of the condition worsen during the clinical trial. During the recording of such events in the AE section of the CRF, it is important to convey the concept that the pre-existing condition has changed by including applicable descriptors (e.g., "more frequent headaches"). Events that are not clearly consistent with the expected pattern of progression of the underlying disease should be recorded as AEs. This includes worsening or relapse of COVID-19 that cannot be attributed to the expected course of disease progression under treatment, taking into account baseline clinical and laboratory findings and the medical history of each participant. These data will be recorded only as efficacy assessment data. In most cases, the expected pattern of progression will be based on clinical results. In rare cases, the determination of clinical progression will be based on symptomatic worsening. However, every possible effort should be made to document progression using objective criteria. If there is any uncertainty as to whether an event is due to disease progression, it should be reported as an AE. The exception from reporting includes events of disease progression with a fatal outcome which are clearly attributable to disease progression.

The occurrence of AEs must be sought by non-directive questioning of the participant at each visit during the clinical trial. AEs may also be detected when they are reported by the participant during or between visits or through physical examination, laboratory test or other assessments. All AEs must be recorded in the CRF.

Investigators will seek safety information, as defined by this protocol, during each contact with the participant. If an AE has been marked as serious in the CRF by the Investigator, the Investigator will be required to fill in the serious adverse event (SAE) form and submit it to the Safety Manager within the timeframes defined by the legislation and the

protocol.

Adverse events and medications will be coded using the current version of MedDRA dictionary and World Health Organization Dictionary (WHO-DD).

17.2. Definition of an Adverse Drug Reaction

An adverse drug reaction is a response to the administration of a medicinal product which is harmful and unintended, with the causal relationship between the drug and the reported AE being a reasonable possibility.

17.3. Reporting Requirements for Severity and Seriousness

An SAE is any AE which:

- results in death,
- is life-threatening,
- requires participant's inpatient hospitalization or prolongation of participant's existing hospitalization,
- results in persistent or significant disability or incapacity,
- is a congenital anomaly or birth defect,

Medical and scientific judgement should be exercised in deciding whether other situations, should be considered serious reactions, such as important medical events that may not be immediately life-threatening or result in death or hospitalisation, but may jeopardize participant's safety or may require intervention to prevent one of the other abovementioned outcomes. Examples of such adverse events are intensive treatment in an emergency room or at home for the management of allergic bronchospasm, blood dyscrasias or convulsions, for which inpatient hospitalisation is not required, as well as drug dependency or abuse.

Any suspected transmission of an infectious agent via a medicinal product is also considered a SAE.

The term "life-threatening" in this context refers to an event in which the participant is at risk of death at the time of the reaction. It does not refer to a reaction which hypothetically might cause death if it was more severe.

The terms "severe" and "serious" are not synonymous. Severity refers to the intensity of an AE [e.g., rated as mild, moderate, or severe, or according to the Common Terminology Criteria for Adverse Events of the National Cancer Institute (NCI CTCAE)]. The event itself may be of relatively minor medical significance (such as severe headache without any further findings).

Severity and seriousness must be independently assessed for each AE recorded in the CRF.

The AE severity grading scale for the NCI CTCAE (version 4.0) will be used for the assessment of AE severity. The below table will be used for the assessment of severity for AEs that are not explicitly mentioned in the NCI CTCAE.

Table 2: AE severity grading scale

Grade	Severity
**1**	Mild: asymptomatic or mild symptoms; clinical or diagnostic observations only or intervention not indicated
**2**	Moderate: minimal, local, or non-invasive intervention indicated; or limiting age-appropriate instrumental activities of daily living [a]
**3**	Severe or medically significant but not immediately life-threatening; hospitalization or prolongation of hospitalization indicated; disabling or limiting self-care activities of daily living [b, c]
**4**	Life-threatening consequences or urgent intervention indicated [d]
**5**	Death related to AE [d]

Note: Based on NCI CTCAE (v4.0), which can be found at: http://ctep.cancer.gov/protocolDevelopment/electronic_applications/ctc.htm

[a] Instrumental activities of daily living refer to preparing meals, shopping for groceries or clothes, using the telephone, managing money, etc.

[b] Examples of self-care activities of daily living include bathing, dressing and undressing, feeding self, using the toilet and taking medications, for participants who are not bedridden.

[c] If an event is assessed as an “important medical event”, it must be reported as a SAE.

[d] Grade 4 and 5 events must be reported as SAEs.

17.4. Causality Assessment

Investigators should use their knowledge of the participant, the circumstances surrounding the event, as well as an evaluation of any potential alternative causes to determine whether an AE is considered to be related to colchicine, indicating "related”, “not related” or "possibly related" accordingly. The following guidance should be taken into consideration:

- Temporal relationship of event onset to the initiation of clinical trial drug.
- Course of the event, considering especially the effects of dose reduction, discontinuation of clinical trial drug, or reintroduction of clinical trial drug (if applicable).
- Known association of the event with the clinical trial drug or with similar treatments.
- Known association of the event with the disease under clinical trial.
- Presence of risk factors in the administration of concomitant drug treatments to the participant, which are known to increase the occurrence of the event.
- Presence of non-treatment-related factors, which are known to be associated with the occurrence of the event.

17.5. Adverse Events Reporting

17.5.1. Immediate reporting requirements from Investigator to Sponsor

Certain AEs require immediate reporting to allow appropriate measures to be taken to address potential new risks associated with the administration of the drug. The Investigator must report such events to the Sponsor immediately. Under no circumstances should reporting take place more than 24 hours after the Investigator becomes aware of the event. SAEs and pregnancy must be reported to the Sponsor within 24 hours from awareness.

Investigators should record all information that can be gathered immediately (i.e., within 24 hours) in the AE page of the CRF and submit the SAE or pregnancy form to the Safety Manager.

The Investigator must report new significant follow-up information for these events to the Safety Manager. New significant information includes the following:

- New signs or symptoms.
- Significant new diagnostic results.
- Change in the AEs outcome including recovery.
- Additional information on the clinical course of the AEs.

17.5.2. Other reporting requirements

AEs that are suspected to be related to medicinal products other than the drugs under clinical trial should be reported by the Sponsor to the Marketing Authorization Holder of the suspected medicinal product, or to the relevant competent authorities via the national spontaneous reporting system as per the local requirements.

17.5.3. Pregnancy reporting

Female participants of childbearing potential should be instructed to immediately inform the Investigator if they become pregnant during the clinical trial or within 7 days following the last dose of the drug. The same applies for partners that have become pregnant from male participants. A pregnancy form in the CRF must be completed by the Investigator immediately (i.e., no more than 24 hours after awareness of the pregnancy) and sent to the Safety Manager.

The Investigator will consult with the Safety Manager, the scientific supervisor, and the Sponsor in order to decide drug discontinuation, he/she will counsel the female, while discussing the risks for the pregnancy and the possible effects on the foetus. No or limited clinical data are available regarding the use of colchicine in pregnant women. Colchicine should not be used during pregnancy, as well as in subjects of childbearing potential without the use of appropriate contraception. Colchicine must be administered during pregnancy only if explicitly required and if the potential benefit justifies the potential risk to the foetus.

Monitoring of the participant or the pregnant partner of a male participant should continue until conclusion of pregnancy. Any AE/SAE associated with the pregnancy (e.g., an event in the foetus, an event in the mother during or after the pregnancy or a congenital anomaly/birth defect in the child) will be reported in the AE section of the CRF. Any abortion should be classified as a SAE (medically significant), recorded in the AE section of the CRF, and reported to the Sponsor immediately (i.e., no more than 24 hours after awareness).

17.5.4. Congenital Anomalies / Birth Defects

Any congenital anomaly/birth defect in a child born to a female participant exposed to colchicine or the female partner of a male participant exposed to colchicine should be classified as a SAE, recorded in the AE section of the CRF, and reported to the Sponsor immediately (i.e., no more than 24 hours after awareness).

17.6. Adverse Event Reporting Period

Investigators will seek information on AEs at baseline, at visits, as well as in all cases of early termination. All AEs, which are subject to the collecting and reporting requirements outlined in this protocol, whether reported by the participant or noted by the clinical trial personnel, should be recorded on the participant's medical history and in the AEs of the CRF.

All pregnancies reported during the clinical trial should be followed until pregnancy outcome.

17.7. Follow-up

The Investigator should make every possible effort to follow all AEs until the final outcome is reported, for example, until the events resolve to baseline health status or are even more improved, are assessed as stable by the Investigator, until the participant is lost to follow-up or until he/she withdraws his/her consent. Follow-up may be performed by phone, fax, electronic mail, or an unscheduled follow-up visit to obtain additional details on the case and outcome (e.g., from hospital discharge summaries, consultant reports, autopsy reports) in order to perform an independent medical assessment of the reported case.

Follow-up information is sent using a new SAE report form stating that this is a follow-up to the previously reported SAEs and providing the date of the original report. Follow-up information should describe whether the event has resolved or continues, if and how it is managed, as well as whether the participant continued or discontinued his/her clinical trial participation.

At the study completion/early termination visit, the Investigator will instruct each participant to report to the Investigator any subsequent AE that could be related or not to the clinical trial treatment. The Investigator must notify the Safety Manager of any death and AE occurring at any time after discontinuation of participant's clinical trial participation. The Safety Manager must also be notified if the Investigator becomes aware of the development of a congenital anomaly/birth defect in a subsequently conceived offspring and fertility-related problems of a participant that has participated in this clinical trial.

The Investigator will report these events directly to the Safety Manager, either by faxing or by scanning and emailing the relative SAE report form using the fax number or email address provided to the Investigators.

18. STATISTICAL ANALYSIS

18.1. Analysis population

All participants receiving at least one dose of colchicine or the indicated treatment for COVID-19 according to the protocol will be included in the statistical analysis (intention to treat).

Participants receiving at least one dose of colchicine or the indicated treatment for COVID-19 according to the protocol will be included in the Safety Analysis Set and will be undergoing safety endpoint analyses.

18.2. Statistical analysis methodology

Analysis of primary objectives will be performed independently upon completion of all necessary assessments required for them. Analysis of secondary and exploratory objectives will be performed after clinical trial completion.

Quantitative (continuous) variables will be summarized using descriptive statistics (mean value, standard deviation, median and range). Qualitative (categorical) variables will be presented as frequencies and percentages (N, %). Any differences between the mean values of the continuous variables will be assessed using parametric statistical tests (e.g. t-test, ANOVA) or non-parametric equivalents (e.g. Wilcoxon signed-rank test, Mann-Whitney Kruskal-Wallis).

All statistical tests will be 2-sided and will be conducted at a significance level α=5%. The exact p values will be reported, even for non-significant results. All analyses will be conducted using a validated statistical analysis programme (e.g. IBM-SPSS 21 and/or R 3.3.2).

AE and SAE coding will be performed by Preferred Term and System Organ Class, based on the current version of MedDRA medical dictionary. Medications will be coded using the current MedDRA dictionary and the World Health Organization Drug Dictionary (WHO-DD).

Statistical analysis will be performed on the totality of all available information and no statistical method for imputation of missing values will be implemented.

18.3. Exploratory analyses

Because of the critical circumstance, it is possible that further exploratory analyses not foreseen by this protocol may be performed after the completion of participants' data recording.

18.4. Required Sample Size

For the clinical phase, in the time-dependent univariate Cox analysis, for a power of 80% with an expected type I error rate (alpha level) of 0.05, a total of approximately 180 participants (i.e., 90 in each group) is required in order to detect a hazard reduction of 50% (hazard ratio 2 for the control group) (35).

For the biochemical phase, in which continuous variables will be compared, much less participants (approximately 60 in total, i.e., 30 in each group) are required.

Because of the extremely critical circumstance due to the COVID-19 pandemic, results will be analysed every 20 participants completing the protocol with the purpose of becoming immediately available to the scientific community in case there are significant findings.

19. DATA HANDLING AND STUDY MONITORING

19.1. Monitoring, data collection and clinical trial management

During the clinical trial, the Investigator will keep complete and accurate information, including participant's history and medical records as well as ICFs signed by the participants. During the clinical trial, the Investigator will maintain the correspondence with competent regulatory authorities and the Sponsor and information regarding participants' screening, enrollment, discontinuation and completion of the clinical trial.

All data/information will be recorded during the scheduled visit by the Investigator, after training, in the CRFs provided by the Sponsor or its representatives. Only his/her authorized personnel will be able to change CRF data.

The CRFs will be legibly completed using a pen with black/blue ink. Any handwritten data correction in the CRF will be made by crossing out the initial value and writing the revised value along with the date and initials of the person making the change. Correction fluid and erasing are not allowed in the CRF. Only authorized site personnel will be recording or changing CRF data.

Investigators should use correct medical terminology/concepts when recording AEs in the CRF section. Colloquialisms and abbreviations should be avoided. Only one AE term should be recorded in the event field of the CRF.

CRFs must be completed during the visits. CRFs will remain at the site until they are reviewed by the Sponsor or the authorized Clinical Research Organization (CRO). Original CRFs will be collected by the Sponsor or the CRO. An identical copy of the complete set of the CRF for each participant will remain at the clinical trial site.

The Sponsor or its authorized representative shall ensure that the clinical trial Investigator understands all the requirements of the research plan and the procedures described in this protocol as well as all his/her responsibilities for its execution as a treating physician. The Sponsor or its authorized representative will be visiting the clinical trial site to ensure compliance with the protocol and to verify the accuracy and completeness of the information and data reported in the CRFs.

Prior to clinical trial initiation, the Investigator is also required to sign a protocol signature page confirming his/her agreement to conduct the clinical trial in accordance with these documents and all the guidance and procedures found in this protocol.

19.2. Quality Assurance

The Investigator must ensure that participants' anonymity will be maintained. The Investigator will maintain a separate list with participants' codes, their names, addresses and phone numbers. The Investigator must provide the Sponsor or its designee with all clinical trial data. All the information regarding the clinical trial is confidential and proprietary to the Sponsor.

The final clinical study report, also including the statistical analysis, will be prepared after completion or termination of the clinical trial. The Sponsor reserves the right to publish and present clinical trial data at scientific meetings, to national and international Regulatory Authorities. The Investigator may not use the results of this clinical trial for publication or presentation without prior authorization by the Sponsor.

It is the responsibility of the Investigator to provide each participant (or his/her acceptable representative) with full and adequate verbal and written information regarding the objectives and procedures of the clinical trial and the possible risks involved. This information must be provided before he/she participates in any clinical trial-related procedure. Participants must be informed about their right to withdraw from the trial at any time. Written participant information must not be changed without prior approval by the Sponsor and the authorities.

Furthermore, it is the responsibility of the Investigator to personally obtain a signed and dated informed consent, from all participants, as well as for all people who conducted the informed consent discussion to sign, prior to the conduct of any clinical trial-related procedure.

Following data clean-up and database lock, the file exported will be transferred to the Biostatistics department for statistical analysis.

The statistical plan executed for the generation of the results, as well as all relevant documents and forms will be electronically archived.

20. REGULATORY AND ETHICS CONSIDERATIONS

20.1. Regulatory approvals

This is an interventional clinical trial requiring approval for its conduct by the National Organization for Medicines (EOF) and the National Ethics Committee (NEC).

Prior to the enrollment of participants in this clinical trial, the protocol and ICF should be additionally reviewed and receive approval by the Institutional Review Board as per the local regulations.

20.2. Ethical conduct of the clinical trial

This clinical trial will be conducted in accordance with the Declaration of Helsinki and the guidance on Good Clinical Practice of the International Conference on Harmonisation (ICH/GCP) the applicable requirements regarding participants' data protection and the international/local guidance on the conduct of clinical trials during the COVID-19 pandemic.

The Investigator will select the participants as per the inclusion and exclusion criteria described in this protocol. All participants or their legal representatives must sign an ICF prior to their enrollment in the clinical trial.

By signing the ICF, the participants or their legal representatives agree to the use of the information and data of their medical records, as these are described in this protocol for the needs and purposes of the clinical trial, unless they voluntarily drop-out or are withdrawn from the clinical study for any reason.

21. STUDY MANAGEMENT CONSIDERATIONS

21.1. Labelling

Labelling of the investigational products will be carried out by the Sponsor or its designee in accordance with the rules of Good Manufacturing Practice (GMP). Labels will be written in Greek and will meet the requirements of the European Union's Guidance on GMP and the Greek regulatory authorities' guidance.

21.2. Storage

All investigational products should be stored according to the instructions of the summary of product characteristics of each product.

21.3. Destruction of investigational products

Investigational products that have not been used upon study completion or termination, have been damaged, have expired, as well as all empty packaging of the investigational products used at the clinic throughout the duration of the participants' hospitalization, will be collected by the delegated personnel members which are designated by the Principal Investigator.

The destruction process will take place in accordance with the regulations of the investigational site.

21.4. Use of information and publications

Data and information collected during this clinical trial will be recorded in the final clinical study report.

At end of the clinical trial, one or more manuscripts for joint publication may be prepared, with collaboration between the investigators and the Sponsor or one of his designees. The Sponsor's scientific supervisor reserves the right to be listed as the last author in all publications concerning this clinical trial.

If the Investigator wishes to independently publish/present any results from the clinical trial, the draft manuscript or presentation must be submitted in writing to the Sponsor for comment and approval prior to the submission of the material for publication/presentation.

21.5. Confidentiality and protection of personal data

Personal data concerning this clinical trial are protected by the Greek Legislation (Law No. 2472/1997, "PROTECTION OF INDIVIDUALS WITH REGARD TO THE PROCESSING OF PERSONAL DATA-INCORPORATING AMENDMENTS, OFFICIAL GOVERNMENT GAZETTE 50/A/10.04.1997).

Participants' personal and medical data will remain confidential. Records related to their participation in the clinical trial, including medical history that may identify the participant and the ICF signed by him/her, will be available for inspection upon request by the EOF/NEC and the Sponsor or its authorized CRO. Through the entire data management, participants' identity will remain confidential. Each participant, upon his/her enrollment in the

clinical trial, will receive an individual participant code, which will be used for the protection of his/her personal data. This number will be used for the identification of the participant throughout the clinical trial and will be used in all clinical trial forms related to the specific participant. The participant number will remain the same throughout the clinical trial, and the assessment and processing of data that will arise from it.

The Sponsor and its representatives are obligated to disclose to the Independent Personal Data Protection Authority the type of files they maintain and how they process the data they collect in the context of the clinical trial activities.

The protocol, the signed ICF, particulars, data and all other information generated will be maintained in strict confidence. No information concerning the inspection or particulars of the clinical trial will be released to any unauthorized third party, without prior written approval by the Sponsor and in accordance with the applicable legislation. Participant medical information obtained from this clinical trial is confidential and may be disclosed to third parties only as permitted by the Informed Consent Form (or separate authorization for use and disclosure of personal health information), which is signed by the participant, unless this is permitted or required by law.

If clinical trial documents must be photocopied during the process of CRF data verification, the participant will be identified by a unique code only; full names/initials and other identifying information will be anonymized.

By signing this protocol, the Investigator also agrees to the handling of all participant data used and disclosed in relation to this clinical trial in accordance with all applicable laws, rules and regulations on personal data protection.

By signing the ICF, the participant accepts being informed of the following:

- The type of personal information (data) that will be collected from participants in this clinical trial.
- The people that have access to the clinical trial information.
- The people that may use or disclose that information.
- The rights of the participant pertaining to the potential revocation of authorization to the use of his/her personal data.

In the event of revocation of a participant’s authorization, the Investigator reserves the right to use the information collected prior to the revocation and efforts should be made to obtain permission to collect all available information at the end of their scheduled clinical trial period.

21.6. Medical records and storage and maintenance of records

The Investigator will ensure that only duly authorized people are delegated with clinical trial-related duties.

During the clinical trial and after termination of the clinical trial, including early discontinuation of the clinical trial, the Investigator must maintain copies of all documents and files relating to the clinical trial conduct.

This documentation includes, but is not limited to, protocols, CRF records, AE reports, patient initial data, correspondence with regulatory authorities and scientific committees, participants' informed consent forms, Investigator's curricula vitae and clinical trial monitor visit logs.

Source documents are original documents, data, and records from which the participant's case report form data are obtained. These include, but are not limited to, hospital records, clinical and administrative charts, laboratory and pharmacy records, diaries, and X-rays. All the information entered in the CRF must be traceable to these medical records in the participant's file. Prior to clinical trial initiation, the types of medical records that contain clinical trial-related information will be clearly defined in the Clinical Trial Monitoring Plan. This includes all protocol data to be entered directly into the CRF (i.e., without any prior written or electronic record of data) and that are considered source data.

The Investigator must also keep the original informed consent form signed by the participant (a signed copy is provided to the participant). The Investigator must provide the clinical trial Sponsor (or designee) access to all relevant medical records to verify their consistency with the CRF entries. Information in medical records relevant to the participants' identity will not be disclosed.

Participant's files and other medical records must be retained for the maximum time period permitted by the hospital/clinic, or as specified below. If the Investigator wishes to assign the records to another person or to transfer them to another location or he/she is unable to retain them for the specified time period, he/she should consult the Scientific Supervisor of the clinical trial.

The Investigator must retain the clinical trial records for the time period specified by the applicable laws and regulations. At a minimum, clinical trial records must be retained for the time period specified by the applicable legislation, i.e., clinical trial records must be retained for at least 25 years after clinical trial completion. These documents may be retained for a longer time period if required by the clinical trial Sponsor and this period and method of retention will be agreed to separately between the clinical trial Sponsor and the clinical trial Investigators. The Investigator must consult with the Sponsor prior to the destruction of the clinical trial and participant records.

If the Investigator transfers his/her registered office, is retired, or for any other reason withdraws from the clinical trial, the Sponsor must be previously notified. Clinical trial records must be transferred to an acceptable designated person, as for example another Investigator, other medical institution, or the Sponsor's designees. Before the Investigator may dispose any records, he/she must have the Sponsor's written permission.

21.7. Training of clinical trial site personnel

The Principal Investigator will ensure that appropriate training relevant to the clinical trial is provided to all the personnel involved, and that any new information relevant to the performance of this clinical trial are forwarded to the personnel involved:

The Principal Investigator of each site will also maintain a record with all people involved in the clinical trial (medical, nursing, and other personnel).

21.8. Protocol and ICF amendments

Protocol amendments may be implemented only after the written approval by the clinical trial Sponsor. In case of a clinical trial protocol or ICF amendment, the required procedure will be followed in accordance with all national regulatory requirements and applicable regulations.

21.9. Early discontinuation of the clinical trial

Both the clinical trial Sponsor and the Investigator may decide to proceed to the discontinuation of a site's participation in the clinical trial, at any time. However, a joint decision regarding the procedures to be followed will be made.

If the clinical trial is early discontinued or suspended, the Investigator will promptly inform the participants and will ensure they are properly treated and followed. Moreover, the competent authorities must be notified in due course and a detailed written analysis must be provided.

21.10. Critical documentation

Prior to clinical trial initiation, the following documents must be collected and archived by the clinical trial Sponsor:

- Curricula Vitae of the Principal Investigators and Co-investigators (recent, signed, dated).
- Signed and dated Investigator's Agreement to the final protocol.
- Signed and dated Investigator's Agreement to any amendments of the clinical trial documents, if applicable.
- Copy of the Institutional Review Board approval letter for sites planned to participate in the clinical trial.
- Financial Agreement/Investigator's Contract.

21.11. Audits and inspections

The Sponsor may perform audits of the clinical trial files in order to ensure compliance with the clinical trial requirements, the guidelines for Good Clinical Practice and the applicable regulatory requirements.

Additionally, the competent authorities may also inspect the sites, at any time during the conduct of the clinical trial or following its completion. In case of an audit or inspection, the Investigator (and the institution) shall agree to allow the auditor(s) and inspector(s) to have direct access to all relevant documents and provide adequate time, to both him/her and his/her personnel, for discussion regarding any findings/major issues.

Participants shall be informed that duly authorized personnel of the Sponsor, or the regulatory authority may inspect their medical records.

Participant's data will remain confidential during the clinical trial and following its completion, and they will not be disclosed to any unauthorized third party.

21.12. Participant Information and Consent

The clinical trial ICF template will be provided to each site in the local language.

The ICF must be signed and dated by the participant (or the participant's legally authorized representative, if applicable) prior to his/her participation in the clinical trial. In case the participant or his/her legally authorized representative is unable to read, then an impartial witness must be present during the discussion regarding the informed consent and the witness must also sign and date the ICF. After obtaining the written informed consent, the person who conducted the informed consent procedure will sign and date the ICF. The case history or clinical records of each participant will document the informed consent procedure and that the written informed consent was obtained prior to participation in the clinical trial.

By signing the ICF, the participant confirms that he/she has been informed about the study and agrees to anonymous data collection, pooling of data with similar scientific data (if applicable), as well as the possibility of clinical trial monitoring activities. It is the responsibility of the Investigator to obtain written informed consent from each person participating in the clinical trial, after appropriate explanation of the aims, methods, anticipated benefits, and potential risks of the clinical trial. The Investigator must also explain that the participant is completely free to refuse to participate in the clinical trial or to drop-out at any time, for any reason without losing the benefit of any medical care to which he/she is entitled or is currently receiving.

The ICF should be revised, whenever there are changes to clinical trial procedures or when new information becomes available that may affect the willingness of the participant to participate.

Participants must re-consent to the most current version of the ICF (or to significant new information/findings addendum in accordance with applicable laws) during their participation in the clinical trial. For any updated or revised ICF, the case history or clinical records of each participant will document the informed consent procedure and that written informed consent was obtained using the updated/revised ICF for the continuation of his/her participation in the clinical trial.

A copy of each signed ICF must be provided to the participant or participant's legally authorized representative and witness, if applicable. All signed and dated ICFs must remain in

each participant's clinical trial file or in the site file and must be available for verification by the clinical trial monitors at any time.

21.13. Completion of the clinical trial

The Institutional Review Board/Board of Directors of the participating hospital sites and competent authorities will be notified of the end of the clinical trial (last participant out) or early discontinuation of the clinical trial, unless otherwise required by the national regulations governing the conduct of such type of studies which may have been altered by the time of clinical trial completion.

21.14. Participants' Insurance

In accordance with the applicable legislation, all participants will be covered against possible health damages as a direct consequence of their participation in this clinical trial.

22. LIMITATIONS OF RESEARCH METHODS

Lack of blinding in the treatment group and in the disposition of placebo versus colchicine introduces participants' selection bias.

For the control and minimization of participants' selection bias, physicians will be invited to enroll consecutively and thus not selectively the first participants (based on the specific target of the site) that are present in their clinic and meet the specific eligibility criteria of the clinical trial.

All concurrent conditions at the baseline visit as well as concomitant medications both at baseline as well as throughout the conduct of the clinical trial will be recorded to examine their potential limiting effect. The possible effect of the limiting factors on the results of this clinical trial will be taken into consideration in the statistical analysis with the use of a strong multivariate analysis, if deemed appropriate.

With regards to the external validity, i.e., the ability of generalization of the results across a more general population, every effort will be made for the clinical trial population to be representative of the overall population of participants with COVID-19 requiring treatment, by enrolling participants from geographically diverse locations across Greece with a non-limiting set of clinical characteristics, except for those indicated by the protocol.

The internal validity of the results will be ensured to the extent that this is feasible by the implementation of appropriate source data verification and quality assurance measures.

The inability to identify the initial time of infection and of becoming ill from SARS-CoV-2 may lead to an inconsistency in participants' infectious and immunological status.

23. REFERENCES

1. Anon. The 2015 ESC Guidelines on the diagnosis and management of pericardial diseases. - PubMed - NCBI. Available at: https://www.ncbi.nlm.nih.gov/pubmed/26547486. Accessed March 25, 2020.

2. Calkins H, Hindricks G, Cappato R, et al. 2017 HRS/EHRA/ECAS/APHRS/SOLAECE expert consensus statement on catheter and surgical ablation of atrial fibrillation: Executive summary. J. Arrhythmia 2017;33:369–409.

3. Hemkens LG, Ewald H, Gloy VL, et al. Cardiovascular effects and safety of long-term colchicine treatment: Cochrane review and meta-analysis. Heart 2016;102:590–596.

4. Tardif JC, Kouz S, Waters DD, et al. Efficacy and safety of low-dose colchicine after myocardial infarction. N. Engl. J. Med. 2019;381:2497–2505.

5. Zhou F, Yu T, Du R, et al. Clinical course and risk factors for mortality of adult inpatients with COVID-19 in Wuhan, China: a retrospective cohort study. Lancet 2020.

6. Ruan Q, Yang K, Wang W, Jiang L, Song J. Clinical predictors of mortality due to COVID-19 based on an analysis of data of 150 patients from Wuhan, China. Intensive Care Med. 2020. Available at: http://www.ncbi.nlm.nih.gov/pubmed/32125452. Accessed March 26, 2020.

7. Grailer JJ, Canning BA, Kalbitz M, et al. Critical Role for the NLRP3 Inflammasome during Acute Lung Injury. J. Immunol. 2014;192:5974–5983. Available at: http://www.ncbi.nlm.nih.gov/pubmed/24795455. Accessed March 25, 2020.

8. Li D, Ren W, Jiang Z, Zhu L. Regulation of the NLRP3 inflammasome and macrophage pyroptosis by the p38 MAPK signaling pathway in a mouse model of acute lung injury. Mol. Med. Rep. 2018;18:4399–4409.

9. Jones HD, Crother TR, Gonzalez-Villalobos RA, et al. The NLRP3 inflammasome is required for the development of hypoxemia in LPS/mechanical ventilation acute lung injury. Am. J. Respir. Cell Mol. Biol. 2014;50:270–80. Available at: http://www.ncbi.nlm.nih.gov/pubmed/24007300. Accessed March 25, 2020.

10. Dolinay T, Kim YS, Howrylak J, et al. Inflammasome-regulated cytokines are critical mediators of acute lung injury. Am. J. Respir. Crit. Care Med. 2012;185:1225–1234. Available at: http://www.ncbi.nlm.nih.gov/pubmed/22461369. Accessed March 25, 2020.

11. Castaño-Rodriguez C, Honrubia JM, Gutiérrez-Álvarez J, et al. Role of Severe Acute Respiratory Syndrome Coronavirus Viroporins E, 3a, and 8a in Replication and Pathogenesis. MBio 2018;9. Available at: http://www.ncbi.nlm.nih.gov/pubmed/29789363. Accessed March 25, 2020.

12. Nieto-Torres JL, DeDiego ML, Verdiá-Báguena C, et al. Severe acute respiratory syndrome coronavirus envelope protein ion channel activity promotes virus fitness and pathogenesis. PLoS Pathog. 2014;10:e1004077. Available at: http://www.ncbi.nlm.nih.gov/pubmed/24788150. Accessed March 25, 2020.

13. Nieto-Torres JL, Verdiá-Báguena C, Jimenez-Guardeño JM, et al. Severe acute respiratory syndrome coronavirus E protein transports calcium ions and activates the NLRP3 inflammasome. Virology 2015;485:330–9. Available at: http://www.ncbi.nlm.nih.gov/pubmed/26331680. Accessed March 25, 2020.

14. Shi C-S, Nabar NR, Huang N-N, Kehrl JH. SARS-Coronavirus Open Reading Frame-8b triggers intracellular stress pathways and activates NLRP3 inflammasomes. Cell death Discov.

2019;5:101. Available at: http://www.ncbi.nlm.nih.gov/pubmed/31231549. Accessed March 25, 2020.

15. Siu K-L, Yuen K-S, Castaño-Rodriguez C, et al. Severe acute respiratory syndrome coronavirus ORF3a protein activates the NLRP3 inflammasome by promoting TRAF3-dependent ubiquitination of ASC. FASEB J. 2019;33:8865–8877. Available at: http://www.ncbi.nlm.nih.gov/pubmed/31034780. Accessed March 25, 2020.

16. Chen I-Y, Moriyama M, Chang M-F, Ichinohe T. Severe Acute Respiratory Syndrome Coronavirus Viroporin 3a Activates the NLRP3 Inflammasome. Front. Microbiol. 2019;10:50. Available at: http://www.ncbi.nlm.nih.gov/pubmed/30761102. Accessed March 25, 2020.

17. Walls AC, Park Y-J, Tortorici MA, Wall A, McGuire AT, Veesler D. Structure, Function, and Antigenicity of the SARS-CoV-2 Spike Glycoprotein. Cell 2020. Available at: http://www.ncbi.nlm.nih.gov/pubmed/32155444. Accessed March 25, 2020.

18. Morales L, Oliveros JC, Fernandez-Delgado R, tenOever BR, Enjuanes L, Sola I. SARS-CoV-Encoded Small RNAs Contribute to Infection-Associated Lung Pathology. Cell Host Microbe 2017;21:344–355. Available at: http://www.ncbi.nlm.nih.gov/pubmed/28216251. Accessed March 25, 2020.

19. Kaksonen M, Roux A. Mechanisms of clathrin-mediated endocytosis. Nat. Rev. Mol. Cell Biol. 2018;19:313–326. Available at: http://www.ncbi.nlm.nih.gov/pubmed/29410531. Accessed March 25, 2020.

20. Stebbing J, Phelan A, Griffin I, et al. COVID-19: combining antiviral and anti-inflammatory treatments. Lancet. Infect. Dis. 2020. Available at: http://www.ncbi.nlm.nih.gov/pubmed/32113509. Accessed March 25, 2020.

21. Cerquaglia C, Diaco M, Nucera G, La Regina M, Montalto M, Manna R. Pharmacological and clinical basis of treatment of Familial Mediterranean Fever (FMF) with colchicine or analogues: An update. Curr. Drug Targets Inflamm. Allergy 2005;4:117–124. Available at: http://www.ncbi.nlm.nih.gov/pubmed/15720245. Accessed March 25, 2020.

22. Chappey ON, Niel E, Wautier JL, et al. Colchicine disposition in human leukocytes after single and multiple oral administration. Clin. Pharmacol. Ther. 1993;54:360–7. Available at: http://www.ncbi.nlm.nih.gov/pubmed/8222477. Accessed March 25, 2020.

23. Molad Y. Update on colchicine and its mechanism of action. Curr. Rheumatol. Rep. 2002;4:252–6. Available at: http://www.ncbi.nlm.nih.gov/pubmed/12010611. Accessed March 25, 2020.

24. Deftereos S, Giannopoulos G, Papoutsidakis N, et al. Colchicine and the heart: pushing the envelope. J. Am. Coll. Cardiol. 2013;62:1817–25. Available at: http://www.ncbi.nlm.nih.gov/pubmed/24036026. Accessed March 25, 2020.

25. Toldo S, Abbate A. The NLRP3 inflammasome in acute myocardial infarction. Nat. Rev. Cardiol. 2018;15:203–214. Available at: http://www.ncbi.nlm.nih.gov/pubmed/29143812. Accessed March 25, 2020.

26. Leung YY, Yao Hui LL, Kraus VB. Colchicine-Update on mechanisms of action and therapeutic uses. Semin. Arthritis Rheum. 2015;45:341–350. Available at: http://www.ncbi.nlm.nih.gov/pubmed/26228647. Accessed March 25, 2020.

27. Marques-da-Silva C, Chaves MM, Castro NG, Coutinho-Silva R, Guimaraes MZP. Colchicine inhibits cationic dye uptake induced by ATP in P2X2 and P2X7 receptor-expressing cells: implications for its therapeutic action. Br. J. Pharmacol. 2011;163:912–26. Available at:

http://www.ncbi.nlm.nih.gov/pubmed/21306580. Accessed March 25, 2020.

28. Misawa T, Takahama M, Kozaki T, et al. Microtubule-driven spatial arrangement of mitochondria promotes activation of the NLRP3 inflammasome. Nat. Immunol. 2013;14:454–460.

29. Martínez GJ, Robertson S, Barraclough J, et al. Colchicine Acutely Suppresses Local Cardiac Production of Inflammatory Cytokines in Patients With an Acute Coronary Syndrome. J. Am. Heart Assoc. 2015;4:e002128. Available at: http://www.ncbi.nlm.nih.gov/pubmed/26304941. Accessed March 25, 2020.

30. Robertson S, Martínez GJ, Payet CA, et al. Colchicine therapy in acute coronary syndrome patients acts on caspase-1 to suppress NLRP3 inflammasome monocyte activation. Clin. Sci. (Lond). 2016;130:1237–46. Available at: http://www.ncbi.nlm.nih.gov/pubmed/27129183. Accessed March 25, 2020.

31. Deftereos S, Giannopoulos G, Angelidis C, et al. Anti-Inflammatory Treatment With Colchicine in Acute Myocardial Infarction: A Pilot Study. Circulation 2015;132:1395–403. Available at: http://www.ncbi.nlm.nih.gov/pubmed/26265659. Accessed March 25, 2020.

32. Anon. WHO | Coronavirus disease (COVID-2019) R&D. Available at: https://www.who.int/blueprint/priority-diseases/key-action/novel-coronavirus/en/. Accessed March 25, 2020.

33. Wang Y, Fan G, Salam A, et al. Comparative Effectiveness of Combined Favipiravir and Oseltamivir Therapy Versus Oseltamivir Monotherapy in Critically Ill Patients With Influenza Virus Infection. J. Infect. Dis. 2019.

34. Cao B, Wang Y, Wen D, et al. A Trial of Lopinavir-Ritonavir in Adults Hospitalized with Severe Covid-19. N. Engl. J. Med. 2020. Available at: http://www.ncbi.nlm.nih.gov/pubmed/32187464. Accessed March 25, 2020.

35. Chow S, Shao J, Wang H. No Title. In: Sample Size Calculations in Clinical Research. 2nd Ed. Chapman & Hall/CRC Biostatistics Series., 2008.

ANNEX

Effective methods of contraception

Women of childbearing potential who are sexually active with a non-sterilized male partner should use at least 1 highly effective method of contraception. Non-sterilized male partners of female participants should use a male condom in combination with spermicide throughout this period. Cessation of birth control after this time point should be discussed with the study doctor. Not engaging in sexual activity for the entire duration of the drug treatment and the drug washout period is an acceptable practice. However, periodic abstinence, the rhythm method and the withdrawal method are non-acceptable methods of contraception. Female participants should also refrain from breastfeeding throughout this period.

- Male participants with a female partner of childbearing potential
  - Non-sterilized male study participants who are sexually active with a female partner of childbearing potential should use a male condom in combination with spermicide. Not engaging in sexual activity is an acceptable practice. However, periodic abstinence, the rhythm method and the withdrawal method are not acceptable methods of contraception. Male participants should refrain from sperm donation throughout this period.
  - Female partners (of childbearing potential) of male study participants should also use a highly effective method of contraception throughout this period.
- Highly effective methods of contraception, defined as those that result in a low failure rate (i.e. less than 1% per year) when used consistently and without errors, are described below. It should be noted that certain methods of contraception are not considered highly effective (e.g. male or female condom with or without spermicide, female cap, diaphragm, or sponge with or without spermicide, non-copper containing intrauterine device, progestogen-only oral hormonal contraceptive pills where inhibition of ovulation is not the primary mode of action [excluding Cerazette/desogestrel which is considered highly effective] and triphasic combined oral contraceptive pills).

Highly Effective Methods of Contraception (Failure Rate <1%):

1) Barrier methods / Intrauterine methods: Copper-containing intrauterine device; levonorgesterel-releasing intrauterine system (additionally considered a hormonal method).
2) Hormonal methods: Etonogestrel implants; intravaginal device (e.g. ethinylestradiol and etonogestrel); medroxyprogesterone injection; normal and low dose combined oral contraceptive pill; norelgestromin/ethinylestradiol transdermal system; desogestrel.
