# Supplementary material for: Effect of Colchicine vs Standard Care on Cardiac and Inflammatory Biomarkers and Clinical Outcomes in Patients Hospitalized With Coronavirus Disease 2019: The GRECCO-19 Randomized Clinical Trial
Source: JAMA Netw Open. 2020 Jun 24;3(6):e2013136. doi: 10.1001/jamanetworkopen.2020.13136 (PMC7315286; doi:10.1001/jamanetworkopen.2020.13136)
Supplement: Supplement 2. — eFigure. Levels of High-Sensitivity Troponin, D-Dimer, and C-Reactive Protein in the 2 Groups eTable. Laboratory Evaluations at Baseline Plus Maximum or Minimum Values [file jamanetwopen-3-e2013136-s002.pdf]

Supplementary Online Content

Deftereos SG, Giannopoulos G, Vrachatis DA, et al; GRECCO-19 investigators. Effect of colchicine vs standard care on cardiac and inflammatory biomarkers and clinical outcomes in patients hospitalized with coronavirus disease 2019: the GRECCO-19 randomized clinical trial. *JAMA Netw Open*. 2020;3(6):e2013136. doi:10.1001/jamanetworkopen.2020.13136

eFigure. Levels of High-Sensitivity Troponin, D-Dimer, and C-Reactive Protein in the 2 Groups
eTable. Laboratory Evaluations at Baseline Plus Maximum or Minimum Values

This supplementary material has been provided by the authors to give readers additional information about their work.

© 2020 Deftereos SG et al. *JAMA Network Open.*

eFigure. Levels of High-Sensitivity Troponin, D-Dimer, and C-Reactive Protein in the 2 Groups

The thick horizontal line indicates the median, the box corresponds to the interquartile range and the whiskers to the range, bar outliers, which are represented by dots. The p values correspond to the Mann-Whitney U test for independent samples.

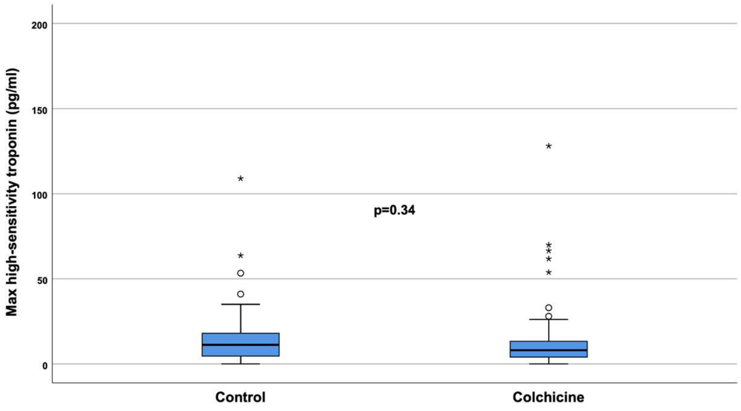

© 2020 Deftereos SG et al. *JAMA Network Open.*

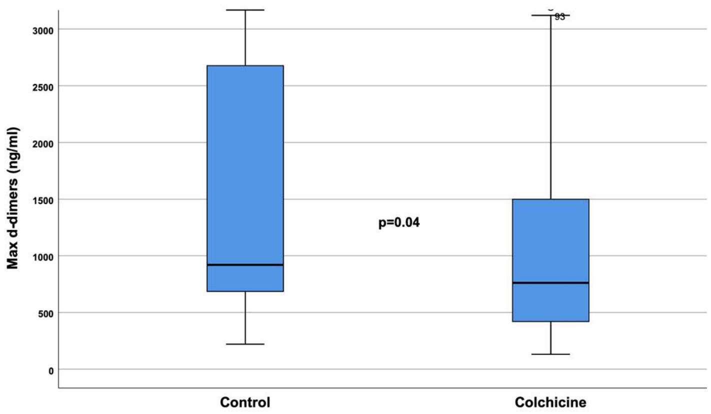

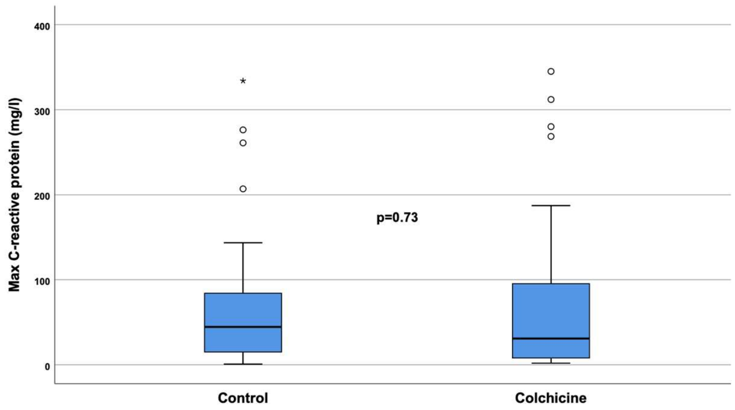

© 2020 Deftereos SG et al. *JAMA Network Open.*

eTable. Laboratory Evaluations at Baseline Plus Maximum or Minimum Values

	**Baseline**				***Maximum or Minimum Value***		
	**Control (N=50)**	**Colchicine (N=55)**	***p***		**Control (N=50)**	**Colchicine (N=55)**	***p***
Temperature - °C	37.7 (37.5 – 38.1)	37.7 (37.5 – 38.1)	0.875	max	36.9 (36.6 - 37.7)	37.0 (36.8 - 37.8)	0.246
Hemoglobin– g/dl	12.8 (11.2 – 14.5)	12.7 (11.5- 14.1)	0.552	min	12.3 (10.8 – 13.6)	11.7 (10.4 – 13.3)	0.572
White Blood Cell count - /µl	5784 (4173 – 8163)	5480 (4820 – 6830)	1.000	max	7189 (5490 - 9040)	6366 (5370 - 8240)	0.148
Neutrophils - %	70.2 (58.6 – 79.9)	67.8 (62.7 – 76.3)	0.871	max	76.9 (66.2 - 80.5)	67.8 (61.8 - 79.1)	0.092
Lymphocytes - %	19.3 (12.6 – 29.2)	23.2 (14.9 – 27.4)	0.778	max	31.6 (21.0 - 38.0)	31.6 (21.8 - 39.3)	0.705
Macrophages - %	8.0 (4.3 – 9.2)	7.6 (6.2 – 8.7)	0.825	max	9.4 (8.0 - 11.7)	10.2 (8.4 - 11.9)	0.332
Eosinophils - %	0.40 (0.15- 1.28)	0.65 (0.18 – 1.80)	0.383	max	2.7 (1.6 – 4.5)	2.5 (1.4 - 3.7)	0.742
Lymphocyte count - /µl	1079 (806-1500)	1299 (899-1608)	0.315	max	920 (676-1293)	1141 (796-1477)	0.109
Platelet count – 10^3/µl	207 (168 – 326)	221 (169 - 327)	0.812	min	213 (148 – 261)	225 (164 – 296)	0.321
Glucose – mg/dl	106 (91 – 126)	100 (88 – 125)	0.543	max	138 (113 – 163)	119 (98 – 194)	0.058
Estimated GFR - ml/min/1.73m²	93 (64 – 118)	99 (76 – 128)	0.149	min	86.7 (55.8-114.3)	100.9 (64.2-120.5)	0.209
Aspartate Transaminase - IU/l	34 (23 – 52)	30 (21 – 42)	0.245	max	48 (29 – 71)	46 (35 – 60)	0.760
Alanine Transaminase – IU/l	35 (18 – 49)	25 (17 – 43)	0.201	max	53 (29 – 88)	56 (35 – 78)	0.997
Lactate Dehydrogenase – IU/l	280 (224 - 405)	251 (196 – 350)	0.160	max	315 (235 - 477)	315 (219 – 415)	0.535
Creatine Phosphokinase – IU/l	80 (55 – 133)	80 (49 – 164)	0.944	Max	107 (55 – 179)	105 (55 – 167)	0.745

© 2020 Deftereos SG et al. *JAMA Network Open.*

C-Reactive Protein – mg/l	40 (12 – 95)	36 (10-67)	0.349	max	45 (14 – 89)	31 (8 – 98)	0.729
hs-troponin – pg/ml	7.0 (3.5 – 18.5)	8.0 (4.0 – 12.3)	0.749	max	11.2 (4.3 – 19.3)	8.0 (4.0 – 13.5)	0.337
D-dimers– ng/ml	596 (395 – 1005)	520 (283 – 939)	0.362	max	920 (678 - 2766)	761 (408 - 1588)	**0.041**

© 2020 Deftereos SG et al. *JAMA Network Open.*
