# Supplementary material for: Effect of Colchicine vs Standard Care on Cardiac and Inflammatory Biomarkers and Clinical Outcomes in Patients Hospitalized With Coronavirus Disease 2019: The GRECCO-19 Randomized Clinical Trial
Source: JAMA Netw Open. 2020 Jun 24;3(6):e2013136. doi: 10.1001/jamanetworkopen.2020.13136 (PMC7315286; doi:10.1001/jamanetworkopen.2020.13136)
Supplement: Supplement 3. — Data Sharing Statement [file jamanetwopen-3-e2013136-s003.pdf]

Data Sharing Statement

Deftereos. Effect of Colchicine vs Standard Care on Cardiac and Inflammatory Biomarkers and Clinical Outcomes in Patients Hospitalized With Coronavirus Disease 2019. *JAMA Netw Open*. Published June 24, 2020. 10.1001/jamanetworkopen.2020.13136

Data
Data available: Yes
Data types: Deidentified participant data
How to access data: spdeftereos@gmail.com
When available: With publication

Supporting Documents
Document types: None

Additional Information
Who can access the data: Researchers whose proposed use of the data has been approved
Types of analyses: Any purpose
Mechanisms of data availability: With investigator support, after approval of a proposal with a signed data access agreement
